# Stage-specific Proteomes from *Onchocerca ochengi*, Sister Species of the Human River Blindness Parasite, Uncover Adaptations to a Nodular Lifestyle*[Fn FN2]

**DOI:** 10.1074/mcp.M115.055640

**Published:** 2016-05-25

**Authors:** Stuart D. Armstrong, Dong Xia, Germanus S. Bah, Ritesh Krishna, Henrietta F. Ngangyung, E. James LaCourse, Henry J. McSorley, Jonas A. Kengne-Ouafo, Patrick W. Chounna-Ndongmo, Samuel Wanji, Peter A. Enyong, David W. Taylor, Mark L. Blaxter, Jonathan M. Wastling, Vincent N. Tanya, Benjamin L. Makepeace

**Affiliations:** From the ‡Institute of Infection & Global Health, University of Liverpool, Liverpool L3 5RF, UK;; §Institut de Recherche Agricole pour le Développement, Regional Centre of Wakwa, BP65 Ngaoundéré, Cameroon;; ¶Institute of Integrative Biology, University of Liverpool, Liverpool L69 7ZB, UK;; ‖Department of Parasitology, Liverpool School of Tropical Medicine, Liverpool, L3 5QA, UK;; **The Queens Medical Research Institute, University of Edinburgh, Edinburgh, EH16 4JT;; ‡‡Research Foundation for Tropical Diseases and Environment, PO Box 474 Buea, Cameroon;; §§Tropical Medicine Research Station, Kumba, Cameroon;; ¶¶Division of Pathway Medicine, University of Edinburgh, Edinburgh EH9 3JT, UK;; ‖‖Institute of Evolutionary Biology, University of Edinburgh, Edinburgh EH9 3JT, UK;; ‡‡‡The National Institute for Health Research, Health Protection Research Unit in Emerging and Zoonotic Infections, University of Liverpool, Liverpool L3 5RF, UK

## Abstract

Despite 40 years of control efforts, onchocerciasis (river blindness) remains one of the most important neglected tropical diseases, with 17 million people affected. The etiological agent, *Onchocerca volvulus*, is a filarial nematode with a complex lifecycle involving several distinct stages in the definitive host and blackfly vector. The challenges of obtaining sufficient material have prevented high-throughput studies and the development of novel strategies for disease control and diagnosis. Here, we utilize the closest relative of *O. volvulus*, the bovine parasite *Onchocerca ochengi*, to compare stage-specific proteomes and host-parasite interactions within the secretome. We identified a total of 4260 unique *O. ochengi* proteins from adult males and females, infective larvae, intrauterine microfilariae, and fluid from intradermal nodules. In addition, 135 proteins were detected from the obligate *Wolbachia* symbiont. Observed protein families that were enriched in all whole body extracts relative to the complete search database included immunoglobulin-domain proteins, whereas redox and detoxification enzymes and proteins involved in intracellular transport displayed stage-specific overrepresentation. Unexpectedly, the larval stages exhibited enrichment for several mitochondrial-related protein families, including members of peptidase family M16 and proteins which mediate mitochondrial fission and fusion. Quantification of proteins across the lifecycle using the Hi-3 approach supported these qualitative analyses. In nodule fluid, we identified 94 *O. ochengi* secreted proteins, including homologs of transforming growth factor-β and a second member of a novel 6-ShK toxin domain family, which was originally described from a model filarial nematode (*Litomosoides sigmodontis*). Strikingly, the 498 bovine proteins identified in nodule fluid were strongly dominated by antimicrobial proteins, especially cathelicidins. This first high-throughput analysis of an *Onchocerca* spp. proteome across the lifecycle highlights its profound complexity and emphasizes the extremely close relationship between *O. ochengi* and *O. volvulus*. The insights presented here provide new candidates for vaccine development, drug targeting and diagnostic biomarkers.

Despite 40 years of vector control and mass drug administration programs, onchocerciasis (river blindness) remains one of the most important of the neglected tropical diseases, infecting nearly 17 million people in sub-Saharan Africa (1). The disease is caused by a filarial nematode, *Onchocerca volvulus*, which resides within subcutaneous nodules and achieves a reproductive lifespan of >10 years (2). An adult female worm releases ∼1000 first-stage larvae (microfilariae, Mf)^1^ per day (3), which migrate to the skin and eyes, ultimately causing onchodermatitis and ocular pathology. Recent estimates of disease burden indicate that 4.2 million people are affected by severe itching, 746,000 experience poor vision and 265,000 individuals are rendered blind, resulting in a total global morbidity of almost 1.2 million years lived with disability (1, 4).

Historically, vector control was implemented on a massive scale, but the mainstay of current control efforts is a single anthelminthic, ivermectin, which suppresses microfilaridermia for several months following a single annual dose but does not kill the adult worms (5). However, resistance to ivermectin may be emerging in West Africa (6, 7), and this drug is contraindicated in individuals heavily co-infected with *Loa loa* (a filarial parasite coendemic in Central Africa) because of the risk of severe adverse events, such as potentially fatal encephalopathy (8). Two potential adulticidal treatments are under evaluation to accelerate elimination efforts: flubendazole, another anthelminthic used primarily for veterinary indications (9); and antibiotics such as tetracycline derivatives, which target the obligate *Wolbachia* endobacteria present in all stages of *O. volvulus* (10). These drugs will have to overcome bioavailability and safety issues (in the case of flubendazole (11)) or undergo a significant contraction in the duration of the regimen (in the case of doxycycline (10)) before they could be implemented on a wide scale. Vaccine development against onchocerciasis has a long history (12), but despite some recent breakthroughs with antigens such as a mutated form of cysteine proteinase inhibitor (13, 14), a vaccine candidate is yet to reach preclinical development.

An equally pressing challenge for onchocerciasis is rapid, sensitive and specific diagnosis of the disease in a format appropriate for rural Africa. The classical method, which is to examine skin snips for Mf, is far from ideal because of its insensitivity, capacity to cause significant discomfort, and the logistics associated with the biosafety of the biopsy punch (15). Immunoassays for *O. volvulus* antibodies are an important tool for monitoring the potential re-emergence of infection following regional elimination (16), but they can only be used in young children because of the longevity of the humoral response. Other diagnostic approaches include the diethylcarbamazine patch test (based on a hypersensitivity reaction in infected individuals (15)) or measuring transmission at the level of the vector by PCR of pooled blackflies (17). However, the desirability of a simple, noninvasive test to determine if an individual harbors one or more viable adult nematodes has spurred the hunt for onchocerciasis biomarkers in body fluids such as urine (18).

Our understanding of filarial genomics and molecular biology has been shaped largely by the publication of the *Brugia malayi* genome (19) and follow-up studies of its transcriptome (20–22), secretome (23–25), and structural proteome (26). This species is geographically restricted cause of lymphatic filariasis in humans, but is also popular as a laboratory model, as it will complete its lifecycle in jirds and will undergo limited development in mice (27). Furthermore, its availability from a central facility in Athens, Georgia, has greatly facilitated genomic and post-genomic studies on this parasite (28). However, *B. malayi* differs greatly from *O. volvulus* in its much shorter lifespan, location of Mf (which circulate in peripheral blood rather than migrating through the skin), and the lifestyle of the adult worms, which are located in the lymphatic vessels rather than nodules (27) and do not become heavily accreted with host material, unlike *O. volvulus* (29). The number of available filarial genomes has expanded recently with the publication of draft assemblies for *Loa* (30), the canine heartworm, *Dirofilaria immitis* (31), and the release of an unpublished *O. volvulus* genome assembly by the Wellcome Trust Sanger Institute (http://parasite.wormbase.org/Onchocerca_volvulus_prjeb513/Info/Index). Nevertheless, RNA and protein expression in *Onchocerca* spp. have yet to be explored in a high-throughput manner.

Here, we utilize the closest relative of *O. volvulus,* the bovine parasite *O. ochengi* (32, 33), to perform the first global expression study of an *Onchocerca* spp. across the major stages of the lifecycle (Fig. 1). For the past two decades, *O. ochengi* has been exploited in its natural host as an advanced screen for drug (34) and vaccine development (35), and has also revealed fundamental insights into the symbiosis between filariae and *Wolbachia* (36, 37). We show that the proteome of *O. ochengi* exhibits both qualitative and quantitative dynamic changes during development and that the protein families undergoing regulation have almost identical orthologs in *O. volvulus*. Furthermore, we quantify several hundred host and parasite proteins in fluid derived from *O. ochengi* nodules (Fig. 1), revealing the presence of a novel vaccine candidate (a 6-ShK-domain protein), homologs of transforming growth factor (TGF)-β, and bovine antimicrobial proteins that probably derive from neutrophils. A key unexpected finding is that many of the protein families exhibiting stage-specific enrichment across the *O. ochengi* lifecycle are associated with mitochondria, suggesting regulation of energy metabolism during development. These data provide a substantial new resource for the development of new vaccine, drug and diagnostic candidates for this chronically neglected disease.

**Fig. 1.**
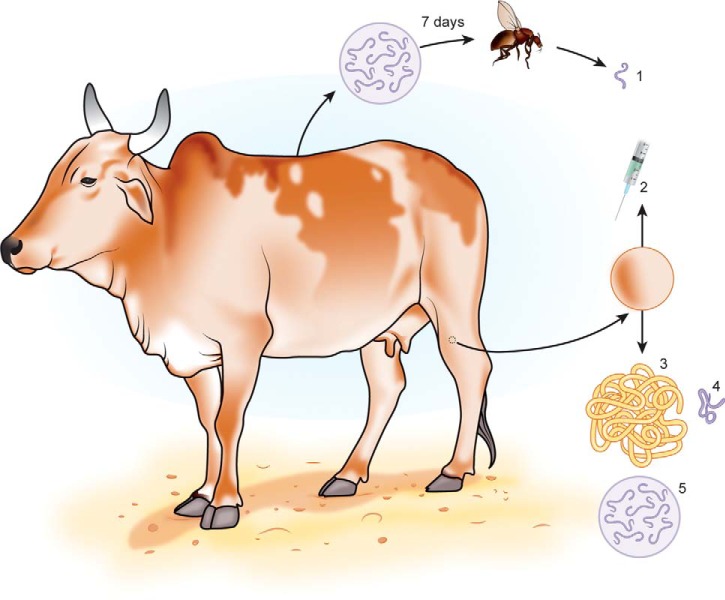
**The lifecycle of *Onchocerca ochengi,* highlighting the sampling strategy of this study.** *Simulium damnosum* blackflies were blood-fed on naturally infected Cameroonian cattle harboring Mf in their skin. The blackflies were reared in the laboratory for 7 days and vL3 (1) were obtained on dissection of the head. To obtain NF, freshly excised intradermal nodules containing adult *O. ochengi* were pricked with a hypodermic needle (2), and the expressed liquid was collected. For the harvesting of adults, ethanol-fixed nodules were digested with collagenase to ensure minimal host contamination on AF (3), whereas AM (4) were obtained directly from freshly dissected nodules. Finally, iuMf (5) were collected from viable AF by puncture of the uteri and harvesting of culture supernatants.

## EXPERIMENTAL PROCEDURES

### 

#### 

##### Parasite Material

Intradermal nodules were excised from the skin of cattle slaughtered at Ngaoundéré abattoir, Adamawa Region, Cameroon, and dissected immediately in PBS to obtain adult males (AM) and adult females (AF; Fig. 1); or were fixed in 50% ethanol. The AM were frozen at −80 °C, while an incision was made in the body wall of viable, gravid AF and the uteri were carefully exposed. The uteri were punctured and the AF were transferred into RPMI medium (supplemented with 200 U/ml penicillin and 200 μg/ml streptomycin) and incubated at 37 °C. Periodically, supernatant fractions were removed from the AF cultures, spun at 300 g for 10 min, and intrauterine microfilariae (iuMf) were counted and assessed for viability (rapid motility). Individual batches displaying >10% sluggish or immotile iuMf were discarded. Following a single wash in PBS, iuMf were frozen as dry pellets at −80 °C. Skin Mf were not collected because of the high prevalence of coinfection with other *Onchocerca* spp. in cattle from this region (38) and the difficulty of reliably separating these species in large numbers. For the collection of vector-derived third-stage larvae (vL3), naturally infected cattle from the Adamawa Region were identified at local markets and transported to the Research Foundation for Tropical Diseases and Environment in Buea. Competent vectors (*Simulium damnosum sensu lato*) were fed on the bait animals, and vL3 were obtained from the blackflies after 7 days (Fig. 1) as previously described (39), except that the vL3 were purified on 20% Percoll gradients and stored at −80 °C in serum-free Grace's Insect Medium (Sigma-Aldrich) without full cryopreservation. Nodule fluid (NF) was harvested from abattoir-derived material as reported previously (40). The AM, iuMf, vL3 and NF were shipped to the UK on dry ice, whereas fixed nodules were transported chilled.

To obtain AF largely free of host tissue, we applied the enzymatic digestion method of Schulz-Key & Karam (41) to ethanol-fixed nodules, which were incubated at 35 °C with orbital shaking at 150 rpm in 0.05% type I bovine collagenase (Sigma-Aldrich). To remove collagenase and host debris, AF were washed in PBS for 2–3 h before any AM were located and discarded. Only young AF that could be liberated from all host tissue after 24 h of digestion were used for proteomic analysis. This reduced the proportion of bovine protein identifications from 70% in AF rinsed in PBS without collagenase digestion, to ∼30% after digestion (data not shown).

##### Protein Extraction

Soluble nematode whole body extracts (WBE) were prepared by adding pooled material for each *O. ochengi* life stage to fresh lysis buffer (40 mm Tris, 6 m urea, 1.5 m thiourea, 66 mm dithiothreitol (DTT, Sigma, St. Louis, MO), complete protease inhibitor mixture (Roche, Penzberg, Germany) and a 1:1 mixture of 1 mm glass and 0.1 mm zirconia-silica beads. Sample were homogenized using a Mini-Beadbeater (Biospec, Bartlesville, OK) for four, 1-min cycles at top speed (with 2-min rest periods on ice between cycles). Samples were centrifuged at 12,000 × *g* for 10 min (4 °C), and both pellet and the supernatant were retained. Protein concentrations were determined using the Pierce Coomassie Plus (Bradford) Protein Assay (Thermo Fisher Scientific, Waltham, MA).

##### Experimental Design and Statistical Rationale

To minimize potential bias and to increase coverage, extracted proteins from each life stage were analyzed using four methodologies. The soluble fraction was subjected to (a) direct tryptic digestion without fractionation, (b) anion exchange fractionation (AEX) of peptide mixtures, and (c) geLC-MS of proteins prior to tryptic digestion. Subsequently, the remaining insoluble pellet was exposed to trypsin to release additional peptides. Furthermore, three analytical replicates of the global (unfractionated) soluble WBE were performed for each life stage. For NF, five biological replicates were analyzed without fractionation in either protein or peptide space. Only protein identifications supported by ≥2 unique peptides present in ≥3 replicate analyses per stage were used for protein abundance comparisons in all cases.

Enrichment of protein domains was assessed using Pfam (EBI, v. 27.0 (42)) as previously described (43) using the gathering threshold as a cut-off. Briefly, a hypergeometric test for enrichment of Pfam domains in the observed proteome (for identifications supported by ≥2 unique peptides only) relative to the complete search database was performed using R (phyper). The Benjamini & Hochberg step-up FDR-controlling procedure was applied to the calculated *p* values (44), and enrichment was considered statistically significant where *p* < 0.01.

##### GeLC-MS

Proteins were fractionated using a NuPAGE® (Thermo Fisher Scientific) precast 4–12% Tris-Bis gradient gel. Each gel lane was cut into 20 slices and in-gel tryptic digestions were performed as described previously by Darby *et al.* (37).

##### Tryptic digestion

Soluble proteins (100 μg) were precipitated by addition of an equal volume of ice-cold 30% (w/v) TCA in acetone with incubation at −20 °C for 2 h. Samples were centrifuged at 12,000 × *g* for 10 min (4 °C) to pellet proteins. Pellets were washed three times with ice-cold acetone and allowed to air dry. Protein pellets were re-suspended in 25 mm ammonium bicarbonate, 0.1% (w/v) RapiGest SF (Waters, Milford, MA). Insoluble material from the initial worm homogenization step was washed three times with 25 mm ammonium bicarbonate and then suspended in 25 mm ammonium bicarbonate, 0.1% (w/v) RapiGest SF. The NF samples were centrifuged at 12,000 × *g* for 10 min (4 °C) and the supernatant retained. The NF protein concentration was determined as described above, and the sample was diluted in 25 mm ammonium bicarbonate, 0.1% (w/v) RapiGest SF. All protein samples were digested as described previously by Armstrong *et al.* (43).

##### Anion Exchange Fractionation

Peptides were fractionated by strong AEX chromatography into four fractions using a method described previously (45). Peptide samples were neutralized with absolute ammonium hydroxide and diluted fourfold in binding buffer (20 mm Britton-Robinson (BR) buffer, pH 11). The peptide mixture was applied to a stack of six layers of Empore anion exchange discs (Agilent) assembled inside a 200 μl pipette tip and centrifuged at 1000 × g for 5 min. The filter was washed with BR buffer and the wash was pooled with the flow-through. Peptides were successively eluted from the anion exchange membrane in 20 mm BR buffer at pH 8, pH 5, and pH 3. Each eluate was desalted and dried as above, before being re-suspended in a 0.1% (v/v) TFA, 3% (v/v) ACN solution for analysis by MS.

##### NanoLC MS ESI MS/MS Analysis

Peptide solutions were analyzed by on-line nanoflow LC using the nanoACQUITY-nLC system (Waters) coupled to an LTQ-Orbitrap Velos (Thermo Fisher Scientific) MS as reported previously (43). The gradient consisted of 3–40% ACN, 0.1% formic acid for 45 min (in-gel digests) or 150 min (nonfractionated soluble and insoluble digests), then a ramp of 40–85% ACN, 0.1% formic acid for 3 min in positive ionization mode.

Peptide samples separated by AEX were analyzed by on-line nanoflow LC using the Thermo EASY-nLC 1000 LC system (Thermo Fisher Scientific) coupled with Q-Exactive mass spectrometer (Thermo Fisher Scientific). Samples were loaded on a 50 cm Easy-Spray column with an internal diameter of 75 μm, packed with 2 μm C_18_ particles, fused to a silica nano-electrospray emitter (Thermo Fisher Scientific). The column was operated at a constant temperature of 35 °C. Chromatography was performed with a buffer system consisting of 0.1% formic acid (buffer A) and 80% ACN in 0.1% formic acid (buffer B). The peptides were separated by a linear gradient of 5–50% buffer B over 240 min at a flow rate of 300 nl/min. The Q-Exactive was operated in data-dependent mode with survey scans acquired at a resolution of 70,000. Up to the top 10 most abundant isotope patterns with charge states +2, +3, and/or +4 from the survey scan were selected with an isolation window of 2.0 Th and fragmented by higher energy collisional dissociation with normalized collision energies of 30. The maximum ion injection times for the survey scan and the MS/MS scans were 250 and 100 ms, respectively, and the ion target value was set to 1e6 for survey scans and 1e4 for the MS/MS scans. Repetitive sequencing of peptides was minimized through dynamic exclusion of the sequenced peptides for 20 s.

##### Protein Identification and Quantification

Spectra were imported into Progenesis QI (v. 2, Nonlinear Dynamics) and processed as described previously (43). Tandem MS data were searched against 13,991 partially revised *Onchocerca* gene models (see *Proteogenomics* below) and those from the *Wolbachia* symbiont, *w*Oo (37) (UniProt release 2014_03; 647 protein sequences); together with predicted proteomes for the bovine host (*Bos taurus*, UniProt release 2014_02; 24,233 protein sequences) and a general contaminant database (GPMDB, cRAP version 2012.01.01; 115 protein sequences). The search parameters were a precursor mass tolerance of 10 ppm and a fragment mass tolerance of 0.8 (LTQ-Orbitrap Velos) or 0.01 Da (Q-Exactive). Two missed tryptic cleavages were permitted. Carbamidomethylation (cysteine) was set as a fixed modification and oxidation (methionine) set as a variable modification. Mascot search results were further validated using the machine learning algorithm Percolator embedded within Mascot. The Mascot decoy database function was utilized and the false discovery rate was <1%, whereas individual percolator ion scores >13 indicated identity or extensive homology (*p* < 0.05). Mascot search results were imported into Progenesis QI as XML files. Relative protein abundance was calculated by the Hi-3 default method in Progenesis (46), in which the abundance of each peptide is calculated from all of its constituent peptide ions and the average abundance of the three top-ranked peptides is used to calculate the protein signal. Protein abundance was normalized across samples by average intensity utilizing the Normalyzer software package (47). *Dirofilaria immitis* excretory-secretory product (ESP) spectral data (MGF files obtained from (48)) were searched against the *D. immitis* theoretical proteome (http://parasite.wormbase.org/Dirofilaria_immitis_prjeb1797/Info/Index; 12,857 protein sequences) using the Mascot (version 2.3.02, Matrix Science) search engine with settings as described above. Data were deposited to the ProteomeXchange Consortium (http://proteomecentral.proteomexchange.org) via the PRIDE partner repository (49) with the data set identifier PXD002889 and DOI 10.6019/PXD002889.

##### Proteogenomics

An unpublished draft *O. ochengi* genome, generated by Gaganjot Kaur and Georgios Koutsovoulos of the Blaxter Laboratory, University of Edinburgh, was downloaded from WormBase ParaSite (http://parasite.wormbase.org/Onchocerca_ochengi_prjeb1809/Info/Index, version nOo.2.0; 13,990 predicted protein-coding genes). The genome assembly was obtained from pooled individuals, and has a Core Eukaryotic Genes Mapping Approach (50) score of 94.4%. However, it is relatively fragmented, comprising 1818 contigs with a N50 length of 12.7 kb. The Wellcome Trust Sanger Trust has released a highly contiguous reference genome for *O. volvulus* (http://parasite.wormbase.org/Onchocerca_volvulus_prjeb513/Info/Index, O_volvulus_Cameroon_v3; 12,143 predicted protein-coding genes; 703 scaffolds with an N50 length of 25.5 Mb) based on sequencing of a single individual, optical mapping, and extensive manual curation (Matthew Berriman and colleagues, unpublished). As split gene models (*i.e.* a single gene incorrectly annotated as two or more genes because of fragmentation between contigs and/or frameshift errors) affect the accuracy of protein identification and quantification, the ProteoAnnotator pipeline (51) was used to objectively assess the strength of evidence for peptide-spectrum matches against *O. ochengi versus O. volvulus* gene models.

The *O. ochengi* gene models from release nOo.2.0 were considered “official” models, whereas *O. volvulus* protein annotations from release WBPS2 were designated as an “alternative” set. In ProteoAnnotator, 642 protein ambiguity groups (PAGs) were identified in which the lead protein was derived from *O. volvulus* rather than *O. ochengi*; thus, these *O. volvulus* gene models were provisionally considered better matches to the MS data and were divided into four groups. In group 1 (*n* = 271), the PAGs contained only *O. volvulus* gene models, presumably because of unassembled regions of the *O. ochengi* draft genome or errors in assembly and prediction. For group 2 (*n* = 273), the lead *O. volvulus* gene model exceeded the length of all *O. ochengi* models in its PAG by ≥10%, which suggests that the *O. ochengi* models were a fragmented subset of the full-length *O. volvulus* model. In group 3 (*n* = 30), the PAGs contained multiple *O. volvulus* and *O. ochengi* gene models and the retained model (from either species) was selected by manual review of the position of unique peptides following alignment of all sequences in the PAG. Finally, in group 4 (*n* = 68), the lead protein was an *O. volvulus* model that was <10% longer than *O. ochengi* models in the same PAG. The search database was revised by adding *O. volvulus* sequences from group 1, replacing existing *O. ochengi* sequences with *O. volvulus* models for group 2, and amending *O. ochengi* models or replacing with *O. volvulus* sequences as appropriate for group 3. For group 4, we took a conservative approach and retained the unmodified gene models from the cognate genome. This amended database contained a total of 13,991 gene models, of which 572 were derived from *O. volvulus*.

##### Bioinformatics

Venn diagrams were created using the Venn diagrams freeware (http://bioinformatics.psb.ugent.be/webtools/Venn), whereas heat-maps were created using GENE-E (http://www.broadinstitute.org/cancer/software/GENE-E) freeware. Functional annotation by Clusters of Orthologous Groups was obtained via the WebMGA server (52). The conserved domain structure of selected proteins was also interrogated in InterProScan 5 (53); sequence alignment was performed using UniProt (Clustal Omega), and sequences were annotated using jalview2 (54). Protein-protein interactions were determined using STRING version 9.1 (55). Prediction of classical N-terminal signal peptides, non-classical secretion signatures, transmembrane domains, propeptide cleavage sites, N-glycosylation sites and mitochondrial targeting signals was performed using the SignalP 4.0 server (56), the SecretomeP 2.0 server (57), the TMHMM 2.0 Server (58), the ProP 1.0 server (59), the NetNGlyc 1.0 server (http://www.cbs.dtu.dk/services/NetNGlyc) and MitoProt (60), respectively. Orthologs to *O. ochengi* proteins in the *B. malayi* (Uniprot release 2013_08; 11,338 protein sequences), *L. sigmodontis* (http://parasite.wormbase.org/Litomosoides_sigmodontis_prjeb3075/Info/Index; 10,246 protein sequences), *D. immitis* (http://parasite.wormbase.org/Dirofilaria_immitis_prjeb1797/Info/Index; 12,857 protein sequences) and *O. volvulus* (http://parasite.wormbase.org/Onchocerca_volvulus_prjeb513/Info/Index; 12,143 protein sequences) theoretical proteomes were determined using reciprocal BLAST (61) with a bit score cut-off of 50.

For phylogenetic analysis of glutathione-domain proteins supported by peptide evidence, *O. ochengi* sequences containing the canonical glutathione transferase (GST) C-terminal and N-terminal domains (Pfam identifiers PF02798 and PF00043, respectively) were obtained from predicted protein-coding sequences available from the WormBase ParaSite. Protein sequences for GSTs from seven species-independent cytosolic GST classes (alpha, mu, omega, pi, sigma, theta, and zeta) and to glutathione-domain containing proteins belonging to the MAPEG (membrane associated proteins in eicosanoid and glutathione metabolism), mPGES2 (membrane-associated prostaglandin E synthase-2), CLIC (chloride intracellular channel protein), EF (eukaryotic elongation factors 1-gamma) and metaxin families were retrieved via BLAST analysis (61, 62) of the NCBI database at http://www.ncbi.nlm.nih.gov (non-redundant GenBank CDS translations, PDB, SwissProt, PIR and PRF, excluding those in env_nr; posted date - Aug 13, 2015 2:33 am). Arthropod and vertebrate glutathione-domain protein sequences were included in the analysis for comparative purposes as representatives of the intermediate and definitive hosts for *O. ochengi* and other filariae. The GST domain-containing protein homologs were subjected to multiple sequence alignment using MUSCLE (63). Phylogenetic bootstrap neighbor-joining trees (subjected to 1000 bootstrap replicates) were produced as PHYLIP output files according to the neighbor-joining method of Saitou & Nei (64) within ClustalX Version 2.1 (65, 66). Default settings for alignments were accepted using the GONNET protein weight matrices with PHYLIP tree format files viewed within the TREEVIEW program (67).

## RESULTS

### 

#### 

##### Overview of protein identifications and comparison with B. malayi

**Fig. 2.**
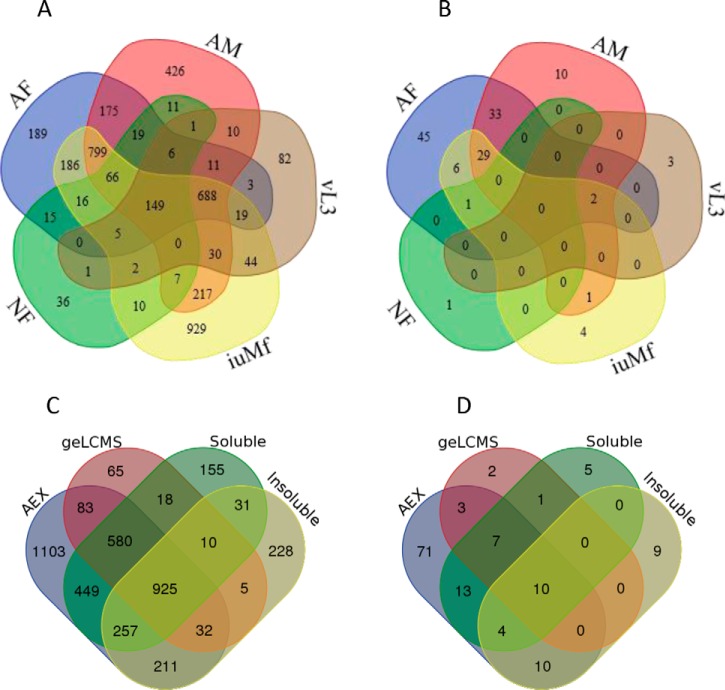
**Venn sets of protein identifications by lifecycle stage and proteomic methodology.** The total number of proteins identified in *O. ochengi* (*A*) and *w*Oo (*B*) in each stage, and the total number of proteins identified in *O. ochengi* (*C*) and *w*Oo (*D*) by methodology. Protein identifications contained in each set are provided in supplemental Table S2.

In this study, we took advantage of the remarkably close relationship between *O. ochengi* and *O. volvulus* to refine our MS search database using a proteogenomic approach based on gene predictions from both species. We identified 4260 unique *O. ochengi* proteins across NF and the four WBE examined, together with 135 proteins from the *Wolbachia* symbiont (*w*Oo), representing 30.4% and 20.8% of the theoretical proteomes, respectively (Fig. 2*A* and 2*B*; supplemental Table S1). Both the greatest total number and largest stage-specific set of protein identifications were derived from iuMf, whereas 16.2% of proteins were shared between all WBE (Fig. 2*A*; supplemental Table S2). The most effective methodology for protein identification, by a wide margin, was fractionation by anion exchange (85.4% of nematode data set), whereas digestion of insoluble pellets uniquely contributed 5.4% of the total (Fig. 2*C* and 2*D*). However, the greatest proportion of predicted transmembrane proteins identified by a single approach (32.9%) was observed by digestion of insoluble pellets (supplemental Table S1). For *w*Oo, 85.9% of all identifications were derived from a single stage (AF) (Fig. 2*B*; supplemental Table S2), which reflects the previously reported dynamics of *Wolbachia* in filarial nematodes and in particular, the large number of bacteria that accumulate in gravid AF during embryogenesis (68, 69). Classification of the detected *w*Oo proteins by Clusters of Orthologous Groups, although purely qualitative (supplemental Fig. S1), clearly reflected the functional allocation of resources in *w*Oo toward translation, post-translational processing and energy conversion, with little representation of coenzyme and secondary metabolism.

**Fig. 3.**
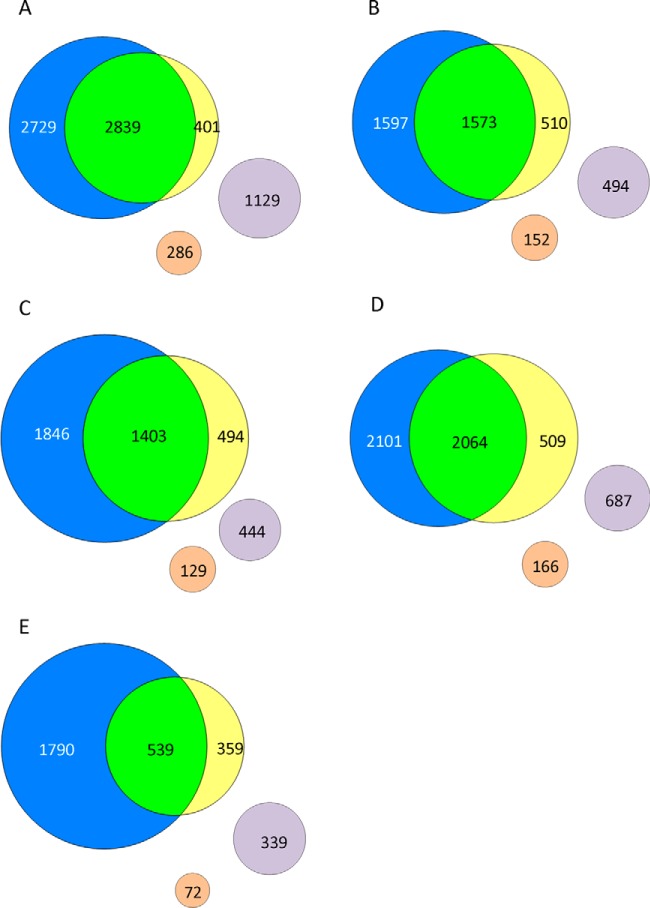
**Venn sets of proteins identified in *O. ochengi* compared with published data for *B. malayi*.** The number of orthologous (blue circles, *B. malayi*; yellow circles, *O. ochengi*) and non-orthologous (lilac circles, *B. malayi*; beige circles, *O. ochengi*) proteins is shown for (*A*) all stages combined, (*B*) AM, (*C*) AF, (*D*) iuMf and (*E*) vL3. Note that the number of proteins per stage is lower than in Fig. 2 because of the exclusion of paralogous gene families. Protein identifications contained in each set are provided in supplemental Table S3.

The only large-scale study of a filarial structural proteome published previously involved analysis of five lifecycle stages of *B. malayi* (the same WBE as the current study, plus mature Mf) by very extensive (∼100 fractions) separation of peptides by strong cation-exchange LC (26). Restricting the comparison to the stages common to both studies, this resulted in the identification of 6697 unique proteins (Fig. 3*A*; supplemental Table S3). Notably, the two studies accorded in attaining the greatest number of protein identifications from iuMf, and the least from vL3 (Fig. 3*D* and 3*E*; supplemental Table S3). Comparisons of orthologous groups revealed that 16.9% of identified *B. malayi* proteins had no ortholog in the *O. ochengi* genome, which was more than double the reciprocal figure (*i.e.* 8.1% of identified *O. ochengi* proteins had no ortholog in the *B. malayi* genome) (Fig. 3*A*). At the genome level, the number of non-orthologous genes in *B. malayi* is only ∼1.4 times greater than the non-orthologous complement in *O. ochengi*, suggesting that Bennuru *et al.* (26) achieved deeper sampling of low-abundance proteins.

To determine whether the protein identifications in each of the shared and unique sets exhibited significant differences, we performed a Pfam domain enrichment analysis between detected proteins and the complete theoretical proteome of *B. malayi* (or *O. ochengi* in the case of nonorthologous proteins from this species). A single domain, “regulators of G protein signaling” (RGS), was overrepresented in the set of *B. malayi*-only orthologs (supplemental Fig. S2). This was because of the detection by Bennuru *et al.* (26) of all six RGS proteins encoded by the *B. malayi* genome, whereas only two of 14 proteins containing RGS domains in the *O. ochengi* genome were identified. Interestingly, collagens were highly enriched in the set of non-orthologous *O. ochengi* proteins, especially those detected in iuMf only, suggesting a gene expansion in this species or loss of some collagen genes from *B. malayi*. However, most enriched domains (“GTP_EFTU_D2”, “pro_isomerase” and proteasome) were shared between the two parasites (supplemental Fig. S2). We conclude that there is no evidence of significant systematic bias between the two studies, although our vL3 data set is probably restricted to the most abundant proteins in this stage.

##### Protein Family Domain Enrichment Analysis

**Fig. 4.**
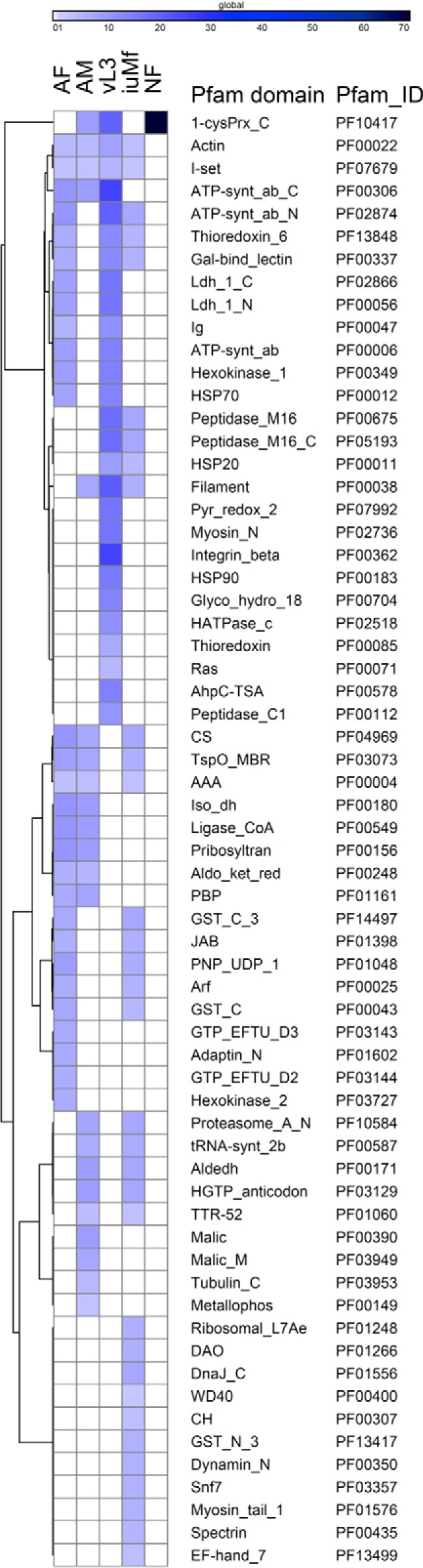
**Pfam enrichment analysis across the lifecycle of *O. ochengi*.** Hierarchically clustered heat-map of protein families that were significantly overrepresented (*p* < 0.01) in the observed proteome (≥2 unique peptides) relative to the complete search database. The intensity of shading is proportional to fold-enrichment.

To identify the protein families that dominated the proteome of each lifecycle stage, we performed an enrichment analysis by comparing Pfam domain counts in observed proteins against those present in all sequences contained in the *Onchocerca* search database. A total of 64 Pfam domains were enriched in one or more lifecycle stages; although only two, actin and I-set domains, were significantly overrepresented in all WBE (*p* < 0.01; Fig. 4). The I-set domain represents the intermediate module from a larger family of immunoglobulin (Ig) domains, which included additional members that were significantly enriched in AF and vL3 only (Fig. 4). In total, 28 proteins were identified with I-set and/or Ig domains, of which 13 were detected in all WBE (supplemental Fig. S3).

We found several members of the pleiotropic β-galactoside-binding protein family (“Gal-bind_lectin” or simply galectin; Fig. 4, supplemental Fig. S4) to be enriched in AF, iuMf and vL3, including almost identical orthologs (nOo_08682 and nOo_10718) of *Ov*-GBP-1 and *Ov*-GBP-2, respectively, which are previously described galectins from *O. volvulus* (70, 71). A more novel finding was the strong representation of Pfam domains associated with intracellular trafficking, including Arf, “Adaptin_N”, Snf7 and WD40, in AF and/or iuMf (Fig. 4, supplemental Fig. S5). However, in a clear example of how the Pfam enrichment analysis accorded with classical biochemical studies of filarial nematodes, vL3 was the only stage displaying significant overrepresentation of enzymes containing “glyco_hydro_18” and “peptidase_C1” domains (Fig. 4). The former group contained chitinases (supplemental Fig. S6*A*), whereas the peptidase domains were found in a group of seven cathepsins (supplemental Fig. S6*B*). Both of these enzyme families are known to be strongly associated with the L3 stage (72–75). A third enzymatic domain specifically enriched in vL3 was the Ras superfamily of small GTPases (Fig. 4, supplemental Fig. S6*C*), which comprises a very large family of regulators and signaling molecules with 56 members in *Caenorhabditis elegans* (76). To the best of our knowledge, Ras proteins have not been reported to be overrepresented in the L3 stage of filarial nematodes previously.

##### Redox and Detoxification Enzymes

**Fig. 5.**
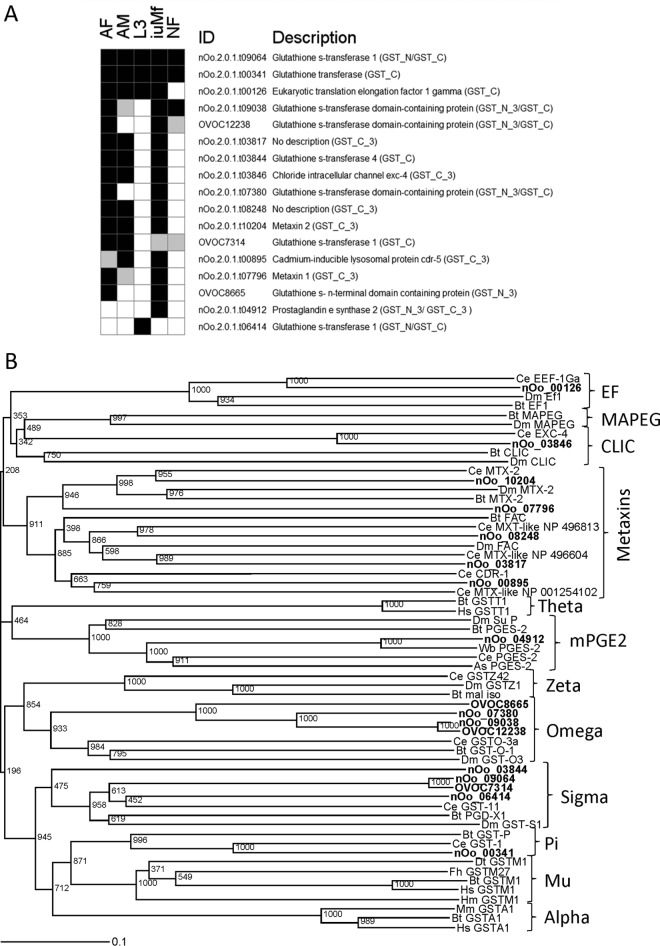
**Detection and phylogeny of GST-domain proteins observed across the *O. ochengi* lifecycle.** *A,* Distribution of GST-domain proteins in each lifecycle stage (black, detected with ≥2 unique peptides; gray, detected with one unique peptide only; white, not detected). *B,* Phylogenetic neighbor-joining tree of GST-domain proteins identified from *O. ochengi* (in bold type) displaying classes present in *C. elegans*, *Drosophila melanogaster, Homo sapiens, Mus musculus,* and selected parasitic nematodes and platyhelminths. Numbers shown alongside branches are bootstrap values of 1,000 replications. Complete accession numbers for all GST-domain sequences are provided in supplemental Table S4. MAPEG, membrane associated proteins in eicosanoid and glutathione metabolism; mPGES2, membrane-associated prostaglandin E synthase 2; CLIC, chloride intracellular channel protein; EF, eukaryotic elongation factor 1-γ.

All stages displayed significant Pfam enrichment for one or more enzyme families with roles in redox reactions and/or detoxification, comprising proteins with thioredoxin, GST or peroxiredoxin (AhpC-TSA/1-cysPrx_C) domains (Fig. 4). Although GST domains were only significantly overrepresented in AF and iuMf, two members of this family were widely distributed across all WBE (Fig. 5*A*). These belonged to two different cytosolic GST subgroups: nOo_00341 within the pi class and nOo_09064 from the sigma class (Fig. 5*B*).

The pi-class GST nOo_00341 is orthologous to OvGST2 that is reportedly the dominant cytosolic GST of *O. volvulus*, abundant in all tissues and life stages (77). In contrast to this single pi-class member, we identified several distinct sigma-class GSTs (Fig. 5*B*), although only nOo_09064 was detected across the entire lifecycle. This GST is homologous to OvGST1 from *O. volvulus,* which has been reported to function as a glutathione-dependent prostaglandin D synthase and is novel among the GST superfamily in containing glycosylated residues (78). The third subgroup of GSTs that have been reported from *O. volvulus* is the omega class, represented by OvGST3, which contains a signal sequence and undergoes alternative splicing (79). This GST is the only member of the superfamily that is significantly up-regulated during exposure of *O. volvulus* AF to oxidative stressors (80). Moreover, OvGST3 exhibits highly restrictive expression in *O. volvulus* adult worms, in that it localizes exclusively to the eggshell of developing embryos from the morula stage onwards (79). Interestingly, although we detected several distinct omega-class GSTs (Fig. 5*B*), they were only robustly identified in AF, iuMf, and (in the case of nOo_09038) NF (Fig. 5*A*). This suggests that these GSTs may be expressed during embryogenesis, although further studies would be required to determine if they are found only in the eggshell.

Two canonical GST superfamily domains typically identify GSTs and reside in the N-terminal and C-terminal regions (PF02798 and PF00043, respectively). In addition to the cytosolic GST forms such as the pi and sigma classes, class-specific C- and N-terminal domains may also occur in other functionally distinct and unrelated proteins that may lack classical glutathione-conjugating activity, including the mPGES2 (81), CLIC (82), EF (83), and metaxin families (84). The single EF family member identified in the current study, eukaryotic elongation factor 1-γ, is essential for delivery of aminoacyl tRNAs to the ribosome and was present in all WBE as expected (Fig. 5*A*). However, we also identified seven proteins that contained a specific GST-like C-terminal domain (PF14497), including five from the metaxin family that were expressed in adult worms and iuMf (Fig. 5*A*). These represent components of the preprotein import complex of the mitochondrial outer membrane; although their function remains obscure (84, 85). The other proteins containing domain PF14497 were prostaglandin E synthase-2 from iuMf only and a CLIC family member, chloride intracellular channel EXC-4, detected in all WBE except vL3 (Fig. 5*A*). The former is a microsome-associated enzyme with a broad specificity for thiol cofactors (81), whereas the latter is essential for the formation and maintenance of correct tubular architecture in the excretory canal of *C. elegans* (86).

Peroxiredoxins (Prx; also known as thioredoxin peroxidases, TPX) are cysteine-dependent antioxidant enzymes that play a major role in the scavenging of reactive oxygen and nitrogen species (87). In *O. volvulus*, Ov-TPX-2 is a member of the Prx1 family of typical 2-Cys Prxs, and is expressed from the late L1 stage in the vector through to adult worms, where it is associated with the body wall, intestine and uterus (88). Embryos and Mf of *O. volvulus* do not express Ov-TPX-2, but are “bathed” in this protein *in utero*, and it can still be detected on the Mf surface after birth (88). The *O. ochengi* Prxs, nOo_08778 and nOo_10285, are almost identical orthologs of Ov-TPX-2, whereas nOo_02155 represents a closely related Prx1 isoform with an N-terminal extension of ∼40 amino acids. In accordance with the *O. volvulus* data, we observed these Prx1 proteins in all WBE, and two isoforms were also significantly enriched in NF (Fig. 4; see *Secreted* Onchocerca *proteins in nodule fluid* below). A fourth Prx1 family member, OVOC4328, was only identified from AM extracts and in common with Bm-TPX-1, contained a predicted N-terminal mitochondrion-targeting domain (89). Indeed, the *C. elegans* ortholog of OVOC4328, PRDX-3, has a key role in the detoxification of mitochondrial hydrogen peroxide generated by the electron transport chain (90). We also identified a single member of the Prx6 family (composed predominantly of 1-Cys members), which is orthologous to OvPXN-2 from *O. volvulus* (91). As reported for *O. volvulus*, this was detected consistently throughout the lifecycle (91).

Significant enrichment for thioredoxin domains was apparent in AF, iuMf and vL3 (Fig. 4), although most of the 14 proteins detected that contained these domains were also identified in AM. Orthologs for two of these proteins (OVOC4952 and nOo_05700) included a protein disulfide isomerase from *O. volvulus* (92) and DPY-11 from *C. elegans* (93), respectively. Both of these proteins are localized to the hypodermal syncytium (as well as iuMf in *O. volvulus*) and are thought to have a role in the catalysis of disulfide bond formation in cuticular collagens (92, 93). Furthermore, an ortholog of OVOC4952 was identified on the surface of AF in *L. sigmodontis*, although not in ESP (43). Two additional proteins detected in all WBE (nOo_01735 and OVOC82) were orthologs of a bi-functional protein disulfide isomerase/transglutaminase from *Dirofilaria immitis* that has been characterized experimentally (94). The transglutaminase activity may be required to form the covalent ε-(γ-glutamyl) lysine isopeptide bonds that are also present in nematode cuticles (94).

##### Mitochondrial Proteins

Mitochondria are dynamic organelles that constantly undergo fission and fusion depending on the energy requirements of the cell and levels of oxidative stress. We identified a number of proteins involved in mitochondrial fission and fusion among the “dynamin_N” Pfam group that was significantly enriched only in iuMf (Fig. 4). These included FZO-1, a “mitofusin” that is required for the formation of tubular mitochondrial structures in the body wall muscle cells of *C. elegans* (95); and EAT-3, a second mitofusin that is essential for optimal ATP production and resistance to damage caused by free radicals (96). Recently, experiments utilizing *C. elegans* mutants have demonstrated a major role for FZO-1 in the rate of growth of L1 and in energy metabolism as measured by oxygen consumption (97). A third dynamin-domain protein identified in iuMf, dynamin-related protein-1, is involved in mitochondrial fission and control of apoptosis (98).

The mitochondrial tRNAs of chromadorean nematodes are unusual in that all lack the typical cloverleaf tRNA structure, with 20 classified as “T-armless” and the remaining two as “D-armless” (99). These tRNAs require specialized elongation factor-Tu proteins, termed TUFM-1 and TUFM-2, to recognize the T-armless and D-armless molecules, respectively. Several elongation factors, eukaryotic translation initiation factors and peptide chain release factors contributed to the significant enrichment of “GTP_EFTU” domains in AF (Fig. 4), most of which were cytosolic and detected in all WBE except vL3. However, a TUFM-1 homolog was also identified in all WBE, whereas a TUFM-2 homolog was restricted to AF and iuMf. The latter binds solely to two D-armless tRNA^Ser^ molecules in chromadorean nematodes (100), and we only detected the mitochondrial form of seryl-tRNA synthetase in iuMf, which contains the Pfam domain “tRNA-synt_2b” (significantly enriched in iuMf; Fig. 4).

Several proteins with pyridine nucleotide-disulfide oxidoreductase (Pyr_redox_2) domains were significantly overrepresented in vL3 only (Fig. 4). These included apoptosis-inducing factor-1 and dihydrolipoamide dehydrogenase, which in healthy cells have roles in redox homeostasis of the respiratory chain and the mitochondrial matrix, respectively (101, 102). A third Pyr_redox_2 domain protein with a mitochondrial-targeting signal peptide was homologous to *C. elegans* thioredoxin reductase (TrxR)-1, a cytosolic enzyme (103). However, the mitochondrial targeting sequence suggests that it is functionally more closely related to *C .elegans* TrxR-2, which is the mitochondrial isoform in this species.

**Fig. 6.**
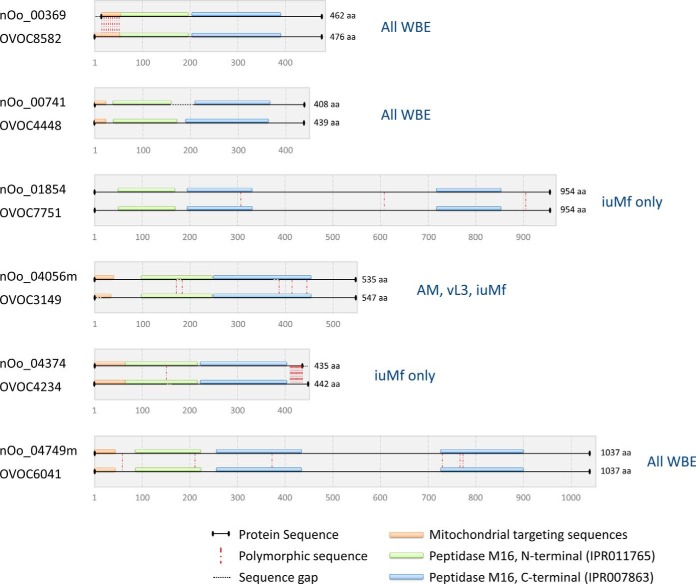
**Domain structure and lifecycle distribution of peptidase family M16 domain proteins.** Schematic representation of *O. ochengi* peptidase family M16 domain proteins and their orthologs in *O. volvulus*. Locus tags containing the suffix “m” refer to modified *O. ochengi* gene models that were manually corrected using *O. volvulus* gene models as a template. Text in blue refers to the lifecycle stages in which the proteins were detected (≥2 unique peptides). Protein signature identifiers from InterProScan 5 (53) are provided for the N- and C-terminal peptidase M16 domains.

Both vL3 and iuMf exhibited enrichment for proteins containing peptidase family M16 domains (Fig. 4), with five and seven members detected with confidence (≥2 peptides), respectively. Four of these (nOo_00369, nOo_00741, nOo_04056, and nOo_04374) resembled core proteins of the cytochrome *bc*_1_ complex or mitochondrial processing peptidase (MPP) (which are closely related (104)). The role of MPPs is to cleave the N-terminal import signal from nuclear-encoded mitochondrial proteins, and the core proteins of the *bc*_1_ complex may function as specialized MPP proteins that target the signal peptide of the Rieske iron-sulfur protein (105); although such peptidase activity has not been demonstrated for *C. elegans* core proteins (104). An additional peptidase family M16 domain protein (nOo_01854), homologous to the mitochondrial presequence protease Cym1, was robustly detected in iuMf only. The role of Cym1 is to degrade the cleaved presequences generated by MPPs in the mitochondrial matrix and release the fragments to the cytosol (106). Because MPPs and Cym1 are also encoded by the nucleus, they should feature an N-terminal mitochondrial targeting sequence. We identified this signal in the *O. ochengi* MPP homologs (Fig. 6), but not in the Cym1 homolog nor in nOo_04749, a further peptidase family M16 domain protein identified in all WBE and annotated as “insulin-degrading enzyme.” However, manual curation of the nOo_04749 gene model with reference to the *O. volvulus* ortholog, OVOC6041, revealed the correct start site and a mitochondrial targeting peptide. This suggests that nOo_04749 is also a MPP, although a highly divergent member of the family (Fig. 6).

##### Quantitative Differences Between Stages

To further explore potential differences in protein expression across the *O. ochengi* lifecycle, we compared protein abundance by a label-free method (Hi-3) for 1313 proteins for which we had detected ≥2 unique peptides across two MS platforms and three sample preparation methodologies (supplemental Table S5). Each lifecycle stage showed a cluster of protein expression containing a variable number of unique proteins (supplemental Fig. S7*A*) that displayed some overlap with the qualitative Pfam enrichment analysis, but was not dependent on tallies of conserved domains. As most proteins derived from *w*Oo were of low abundance, only 13 endosymbiont products fulfilled our criteria for quantification by Hi-3. The pattern of protein expression was clearly related to the known dynamics of *Wolbachia* replication as determined for *B. malayi*, in which Mf and L3 have the lowest bacterial numbers, AM harbor an intermediate level, and gravid AF contain the largest bacterial population (68) (supplemental Fig. S7*B*).

##### Secreted Onchocerca proteins in nodule fluid

**Fig. 7.**
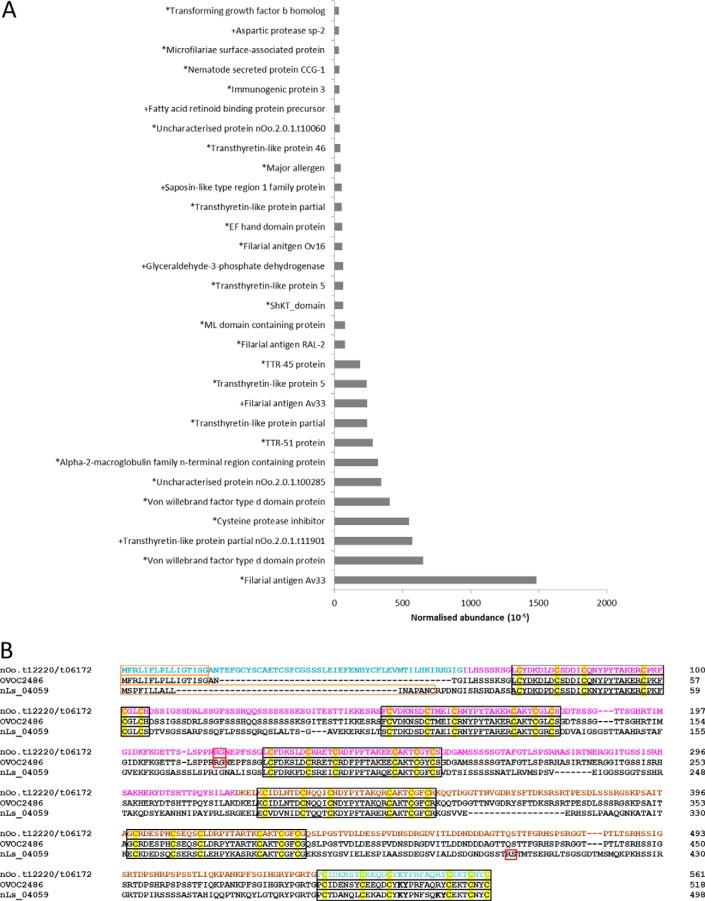
**Abundant *O. ochengi* proteins in nodule fluid and structure of a ShK-domain protein.** *A,* Proteins in NF were quantified by the Hi-3 method and ranked by abundance; the top 30 (from a total of 94 robustly identified) are shown. An asterisk indicates prediction of a classical signal peptide, whereas a plus sign demarks proteins predicted to be secreted by a non-classical pathway. *B,* Reconstruction of a full-length, six ShK-domain protein in *O. ochengi* using the *O. volvulus* gene model OVOC2486 as a template, and alignment against the *L. sigmodontis* ortholog nLs_04059. Highlighted features comprise predicted signal peptides (orange boxes), ShK toxin-like domains (black boxes), conserved cysteine residues (yellow shading), predicted propeptide cleavage sites (red boxes), and lysyltyrosine dyads (bold type). Residues in different colors represent translations from separate contigs.

In NF, we identified 94 O. *ochengi* and 498 bovine proteins with ≥2 unique peptides in ≥3 biological replicates. As expected, the most abundant filarial proteins in NF all featured classical or non-classical secretion signatures (Fig. 7*A*). The ESP repertoire from NF displayed remarkable parallels with that of *L. sigmodontis* gravid AF, with shared orthologs for abundant transthyretin-like and von Willebrand factor type-d domain proteins (43), cysteine proteinase inhibitors (107), and poorly characterized filarial antigens (Av33 (108), Ov16 (109), and RAL-2 (110)). In addition, an uncharacterized NF protein (nOo_00893) had a ML (lipid-binding) domain and was orthologous to an abundant secreted *L. sigmodontis* protein sharing this same motif (43). However, the most important similarity between the NF and *L. sigmodontis* secretomes was the presence of a ShK domain protein (43), which is related to the metridin-like cnidarian toxins that can block Kv1.3 potassium channels in memory T-cells (111) (Fig. 7*B*). Recently, a synthetic C-terminal ShK domain peptide from a *B. malayi* astacin was demonstrated to exhibit Kv1.3-channel-blocking activity for human T-cells, albeit with lower potency than cnidarian ShK peptides (112). In *L. sigmodontis,* the most abundant secreted ShK-domain protein (nLs_04059) is not an astacin and is unusual in containing six ShK domains. We detected several peptides that matched to an *O. volvulus* ortholog (OVOC2486) of nLs_04059, and this sequence enabled us to reconstruct the *O. ochengi* ortholog from two split gene models (nOo_12220 and nOo_06172) covering the central portion along with the N- and C-terminal domains located on other contigs. With the exception of a longer N-terminal region immediately downstream of the signal peptide, the combined “nOo_12220/nOo_06172” gene model is identical to OVOC2486 (Fig. 7*B*). The proteins from both *Onchocerca* spp. exhibit a single lysyltyrosine dyad in the sixth ShK domain (as opposed to two in nLs_04059), which is essential for Kv1.3 channel-blocking activity (111). It is unclear whether cleavage of the sixth ShK domain (the only one containing a LysTyr dyad) from the remainder of the molecule is required for activity *in vivo*, because although nLs_04059 has a propeptide cleavage site upstream of the sixth domain, the only predicted cleavage site within the *Onchocerca* proteins is located in the middle of the molecule (Fig. 7*B*).

The high level of concordance between the NF and *L. sigmodontis* secretomes was maintained when the complete NF dataset was compared with those from other published filarial secretome studies (supplemental Fig. S8). Remarkably, only three proteins were observed in every study, including triosephosphate isomerase, which has recently been shown to have an essential role in Mf production in *B. malayi* (113). As noted by us previously (43), the three *B. malayi* secretome studies display a limited degree of overlap, highlighting the potential impact of methodology on proteomic data; although we observed a strong correlation between our NF dataset and that of Moreno & Geary (24) for *B. malayi* (supplemental Fig. S8). Seven proteins were shared uniquely between the NF, *L. sigmodontis* and *D. immitis* secretomes, including orthologs of nematode secreted protein 22U (114), a cyclophilin (23), and a single transthyretin-like protein.

**Fig. 8.**
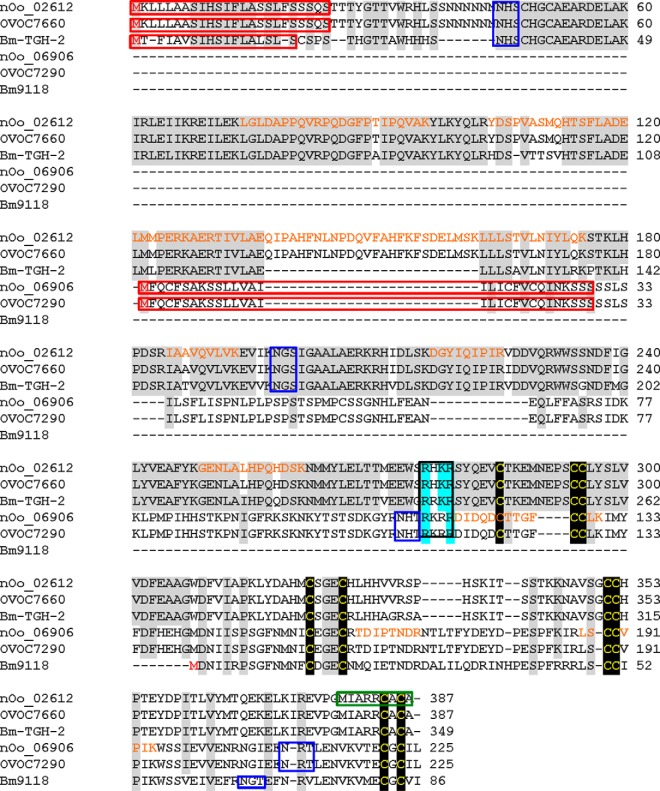
**TGF-β homologs detected in *O. ochengi* nodule fluid.** Alignment of *O. ochengi* TGF-β homologs from NF against orthologs from *O. volvulus* and *B. malayi*. Highlighted features comprise predicted signal peptides (red boxes), start methionines (red type), tetrabasic protease cleavage site (cyan shading), conserved cysteines (yellow type and black shading), potential N-glycosylation motifs (blue boxes), and peptides detected by MS (orange type). The green box represents the corrected C terminus of nOo_02612.

A striking feature of NF, which has not been observed previously in high-throughput secretome analyses of filarial nematodes, was the presence of two TGF-β homologs (Fig. 8). The TGF-β superfamily has members present in all multicellular organisms, and is involved in development, regulation of the mammalian immune response and immunomodulation by parasites. The prototypic TGF-β family member, human TGF-β1, has an N-terminal moiety composed of a signal peptide, a proregion and a protease cleavage site (RXXR), targeting of which releases the C-terminal active domain capable of binding to the receptor. The active domain of TGF-β1 contains nine cysteines necessary for correct folding and dimerization (115).

The first TGF-β family member we identified, nOo_02612, has an identical ortholog from *O. volvulus*, OVOC7660, and is closely related to a *B. malayi* TGF-β family member, *Bm*-TGH-2. Although the predicted sequence of nOo_02612 showed a C-terminal extended region, lacking the CXC motif characteristic of TGF-β family members, closer inspection revealed that this was because of miscalling of an intron-exon boundary (Fig. 8). The second TGF-β family member detected in NF, nOo_06906, also has an identical *O. volvulus* ortholog, OVOC7290, and displays significant homology to another *B. malayi* protein, Bm9118 (Fig. 8). However, Bm9118 lacks the protease cleavage site and first three cysteines of the TGF-β family active domain, suggesting that the gene model may be incomplete. All peptides detected in NF from nOo_02612 were contained in the proregion of the protein, whereas all peptides identified from nOo_06906 were located in the active C-terminal domain (Fig. 8), indicating that nOo_06906 at least is present in secretions in its active form.

As reported above (see *Redox and detoxification enzymes*), the repertoire of Prxs in NF (one Prx6 and two Prx1 isoforms) overlapped with those detected in all WBE, although a single Prx1 observed in all of the structural proteomes (nOo_02155) was not found in NF. This was orthologous to a molecule identified on the surface of *L. sigmodontis* (TPX-1, nLs_01344) that was also not secreted (43). Unexpectedly, nodule fluid also contained six filarial immunoglobulin-domain proteins (supplemental Fig. S3) that are known to localize to extracellular matrices in *C. elegans*. One of these proteins (OVOC10067) was homologous to the perlecan-like proteoglycan UNC-52; a major component of the basement membrane of contractile tissues, including the pharynx and anus, in developing embryos and subsequent stages of *C. elegans* (116). In addition to its structural role, UNC-52 is required for control of directionality during axon outgrowth in *C. elegans* (117); whereas another immunoglobulin-domain protein detected in NF, hemicentin, is involved in the anchoring of mechanosensory neuron axons (118). Furthermore, a peroxidasin homolog related to *C. elegans* PXN-2 was present in iuMf and NF, and this was orthologous to a *L. sigmodontis* peroxidasin that was observed in the secretome of AF and immature Mf (43). In *C. elegans*, PXN-2 is located in the extracellular matrix and is required for late embryonic elongation, muscle attachment, and motoneuron axon guidance choice (119). Finally, a surprising component of NF was a giant (predicted molecular weight, ∼690 kDa) immunoglobulin-domain protein homologous to *C. elegans* DIG-1 (supplemental Fig. S9), the largest secreted protein from any organism described to date (120).

##### Bovine Proteins in Nodule Fluid

**Fig. 9.**
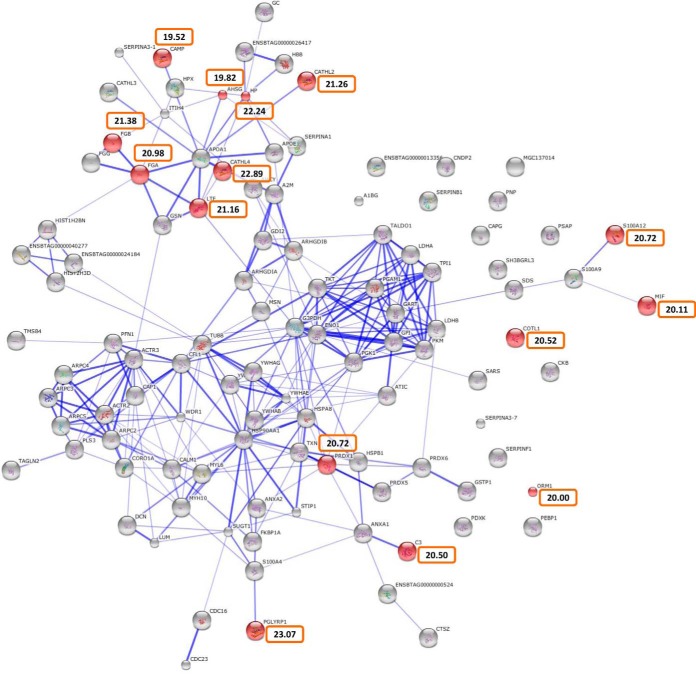
**Protein interaction network of the most abundant bovine proteins in nodule fluid.** The 100 most abundant bovine proteins in nodule fluid, as determined by the Hi-3 method, were subjected to network analysis. The significantly enriched GO term “defense response” (FDR-corrected *p* < 0.01) is highlighted by the red nodes, and orange boxes contain normalized (log_2_) abundance values. CAMP, cathelicidin-7; CATHL2, cathelicidin-2; CATHL4, cathelicidin-4; FGA, fibrinogen alpha-chain precursor; FGB, fibrinogen beta-chain precursor; AHSG, alpha-2-HS-glycoprotein; HP, haptoglobin precursor; LTF, lactotransferrin; MIF, macrophage migration inhibitory factor; COTL1, coactosin-like protein; PRDX1, peroxiredoxin-1; ORM1, alpha-1-acid glycoprotein precursor; C3, complement C3 preproprotein; PGLYRP1, peptidoglycan recognition protein-1.

In addition to serum components, the bovine proteins quantified in NF displayed a high abundance of molecules involved in innate immunity. The predominant 100 bovine proteins were analyzed for interaction networks and GO term enrichment (Fig. 9). The most numerous host cell type in both *O. ochengi* and *O. volvulus* nodules is neutrophils, which form a dense layer around the adult nematodes without causing any apparent damage to the parasites (36, 121, 122). Accordingly, several proteins known to be abundant in bovine neutrophils were significantly enriched within the GO term “defense response” (FDR-corrected *p* < 0.01), including cathelicidin-2 (bactenecin-5), cathelicidin-4 (indolicidin), cathelicidin-7 (bovine myeloid antimicrobial peptide-34) (123, 124), haptoglobin (125),PGLYRP1 (peptidoglycan recognition protein (126)), and S100A12 (calgranulin C) (127) (Fig. 9). All of these proteins are major constituents of neutrophil granules except for calgranulin C, which is cytosolic. This has been purified from extracts of collagenase-treated *O. volvulus* (128), suggesting a tight association with the parasite surface, and has some antifilarial activity, especially against Mf (129).

## DISCUSSION

This study represents the first expression analysis of an *Onchocerca* spp. across the major lifecycle stages and at the immediate host-parasite interface of the nodule. A recurring theme of our analyses was the remarkable similarities between protein families identified by unbiased, proteomic methods in *O. ochengi* and those reported from *O. volvulus* as highly expressed according to classical approaches, such as representation in cDNA libraries from each stage. However, other discoveries emerging from this work, such as the differences in mitochondrion-related protein families dominating in each stage, were not anticipated. Moreover, quantification of nodule fluid proteins provided new insights into the immunomodulatory milieu surrounding the adult worms.

Bennuru *et al.* (26) achieved deeper coverage of the *B. malayi* proteome than we experienced in our study of *O. ochengi.* In all likelihood, this can be attributed to the fractionation protocol they employed. However, other factors may have contributed, such as the manual curation that accompanied the publication of the *B. malayi* genome (19), the utilization of a *B. malayi* EST dataset in addition to gene predictions from the genome (26), the application of a less stringent FDR (2% *versus* 1% in the current study), and the host contamination in *O. ochengi* samples that is more challenging to minimize than is the case for *B. malayi* (because of the nodular lifestyle of the former; see *Experimental Procedures*). In addition, there may be intrinsic differences in the dynamic range of protein expression between the two parasites; or a greater proportion of *O. ochengi* proteins may only be expressed in stages that were outside the scope of this study (mature Mf, L2 and L4). In general, the relative distribution of orthologous and non-orthologous proteins detected in each study across the lifecycle was similar, although vL3 was a significant outlier. This may reflect the challenges of obtaining *O. ochengi* vL3 in significant numbers for analysis, or fundamental differences in the complexity of the infective stage between the two species.

Proteins containing GST domains constituted one of the largest families that were enriched in AF and iuMf. The GSTs are a superfamily of multifunctional proteins that are ubiquitous across animals, plants and bacteria and are much studied in their cytosolic form because of their importance as phase II detoxification enzymes (130). In addition to detoxification, where typically they enzymatically conjugate glutathione with electrophilic, lipophilic, and nonpolar compounds including xenobiotics, GSTs have been reported to carry out a range of other functions. These include binding and transport of hydrophobic ligands (131); synthesis of eicosanoids with roles in immune modulation (132, 133); catabolism of amino acids (134); and inhibition of the Jun N-terminal kinase signaling pathway, thus protecting against hydrogen peroxide-induced apoptosis (135). Proposed functions of OvGST2 include the neutralization of lipid peroxidation products arising from immune-mediate damage to the parasite (77, 136). Conversely, OvGST1 is located in the outer hypodermal lamellae and cuticle, and is released in ESP from AF, suggesting it may have an immunomodulatory role at the host-parasite interface via the generation of prostanoids (137). Thus, our detection of an OvGST1 ortholog (nOo_09064) in NF is likely to reflect an equivalent function for this secreted GST in *O. ochengi.*

A striking feature of our study was the number of mitochondria-related proteins that were overrepresented in different stages, particularly in iuMf and vL3. Notably, it has been known for decades that most adult filariae depend primarily on fermentation of glucose for their energy requirements, whereas Mf also use oxidation of pyruvate obtained from catabolism of glucose or amino-acids (138, 139). The importance of aerobic respiration in iuMf is likely to place significant demands on mitochondrial function. Thus, as expected for an enzyme involved in protection of mitochondria from oxidative stress, deletion of the *trxr-2* gene, which is mainly expressed in body wall and pharyngeal muscles in *C. elegans*, led to delayed development and reduced longevity under stress conditions (103). Moreover, inhibition of TrxR in a filarial parasite of cattle, *Setaria cervi*, impaired the motility and viability of adult worms and iuMf, and was associated with oxidative damage to lipids and proteins, leading to mitochondrial-mediated apoptosis (140). In *B. malayi*, many transcripts encoding mitochondrial metabolic enzymes were up-regulated in vL3 relative to L3 cultured *in vitro* for 2 days (20). During development in the insect vector, the transcriptional profile of *B. malayi* suggests that it primarily uses anaerobic dismutation of malate for its energy needs (22, 141), which occurs in the mitochondrial matrix (142). This process may be associated with an increase in the importation and processing of Krebs cycle enzymes in mitochondria, or vL3 may be “primed” to shift to oxidative phosphorylation for the energy-expensive processes of skin penetration and migration to the lymphatics in the definitive host (20). In this context, it may be informative to examine the interplay of mitochondria and *Wolbachia* during L3 to L4 development, as the latter replicate rapidly during this phase (68) and display high levels of expression for respiratory chain components (37).

Several of the mitochondrial proteins identified in our study have been proposed as drug targets, such as the nematode MPPs, which are poorly conserved relative to their mammalian counterparts (104). Moreover, the divergent TUFMs of nematodes, which exhibit unique C-terminal extensions, constitute attractive targets for highly specific anthelminthics (99). Recently, the gold-containing compound auranofin was identified as a lead adulticidal drug candidate that specifically inhibits filarial TrxR. Importantly, auranofin-treated AF of *O. ochengi* were found to be depleted of mitochondria (143).

A key discovery in NF was the presence of two TGF-β homologs. *Bm*-TGH-2 is a TGF-β family member that can be detected by Western blot in *B. malayi* ESP and can bind and signal through the mammalian TGF-β receptor (144). As TGF-β signaling can inhibit immune responses, suppressing T-cell proliferation and inducing regulatory T-cells, secretion of this filarial homolog has been proposed to have immunomodulatory effects. However, subsequent to the original identification of *Bm*-TGH-2, no proteomic analyses of *B. malayi* ESP have identified secreted TGF-B family members (23–25). In *O. volvulus* and *O. ochengi*, TGF-β family members were reported to be expressed in the basal layer of the cuticle, the intestine, and the reproductive tracts of male and female adult worms, including in the uterine and vaginal muscles, but no attempt to measure secretion was undertaken (145). In contrast, ESP from the gastrointestinal nematodes *Heligmosomoides polygyrus* and *Teladorsagia circumcincta* both contain TGF-β mimics that can signal through the mammalian TGF-β receptor, inducing regulatory T-cells *in vitro* (146). Although *H. polygyrus* also transcribes a TGF-β family member (147), this has been proposed to have a developmental role, and could not be detected in proteomic studies of *H. polygyrus* ESP (148, 149). Although it is unknown whether the *O. ochengi* TGF-β homologs identified here can signal through the mammalian TGF-β receptor in a similar manner to *Bm*-TGH-2, or simply have an internal developmental role associated with “spill-over” into the mammalian host, their presence in NF is suggestive of an active immunomodulatory function.

An initial analysis of the *w*Oo proteome has been published, in which 122 proteins were identified from AF (37). The limited number of additional identifications achieved in the current study was anticipated, because the published analysis was based on a semipurified sample of bacteria, whereas no biological fractionation of *O. ochengi* was attempted here and *Wolbachia* proteomes have a substantial dynamic range (150). A comparison of the *w*Oo proteome with that of its counterpart in *B. malayi* has been explored previously (37). It has been postulated from proteomic data obtained from *Wolbachia* within *B. malayi*, which was dominated by single-peptide identifications as in our study, that the endosymbionts exhibit extensive stage-specific expression during filarial development (26). Considering the stochastic nature of peptide identifications from very low abundance proteins, we find this scenario unlikely and in any case conflated by the substantial changes in *Wolbachia* density throughout the filarial lifecycle.

On the basis of the quantities of bovine antimicrobial proteins observed in NF, *Wolbachia* appears to be a major driver of innate immune responses in the nodule. Depletion of *Wolbachia* endosymbionts with antibiotics leads to a decline in neutrophilia in *O. ochengi* nodules and an influx of eosinophils, which degranulate on the parasite cuticle and ultimately kill the adult nematodes (36, 122). *Wolbachia* proteins that are known to activate neutrophils include WSP (151) and PAL (152), and GroEL may also have a role as there are precedents in other bacteria (153). Surprisingly, and in contrast with AF ESP from *L. sigmodontis* (43), we did not consistently detect *Wolbachia* proteins in NF. However, because WSP has been detected on the surface of *B. malayi* (154) and *L. sigmodontis* (43), symbiont proteins on the cuticle (rather than secreted products) may be a more important trigger for neutrophil activation in onchocerciasis. Both cathelicidins (124) and PGLYRP1 (155) have potent antibacterial activity, and might be liberated by neutrophils following activation of Toll-like receptors 2 and 4 by WSP (156). Importantly, although *Wolbachia* lacks a peptidoglycan-based cell wall (157), PGLYRP1 has been reported to kill other pathogens that lack peptidoglycan (158).

## CONCLUSIONS

We have revealed a remarkable complexity and dynamism of the *O. ochengi* proteome throughout the major lifecycle stages, particularly with respect to mitochondrion-associated proteins, as well as the presence of TGF-β homologs in the *O. ochengi* secretome. In addition, the developmentally regulated protein families that have been identified in *O. ochengi* are represented by almost identical orthologs in *O. volvulus*, suggesting that their patterns of expression will be conserved in the human parasite. Although L3 have historically been the main focus of attention for vaccine design, our data on the overrepresentation of the peptidase M16 family suggests that it may be possible to design drugs to target this stage; whereas another potential source of vulnerability for both immunoprophylactic and chemotherapeutic approaches is embryogenesis. Indeed, mutated forms of cysteine proteinase inhibitor are highly effective at reducing female worm fertility in *B. malayi* (14) and microfilaraemia in *L. sigmodontis* (13), and immunologically targeting of triosephosphate isomerase can also impede embryogenesis in *B. malayi* (113). The evaluation of the ShK-domain protein as a vaccine in animal models is now a priority, and other components of the nodule secretome may constitute promising new diagnostic biomarkers if they can be shown to reach the peripheral circulation. In conclusion, the *O. ochengi* system in cattle will continue to play a critical role in our understanding not only of *O. volvulus* biology, but the means to its ultimate eradication.

## Supplementary Material

Supplemental Data

## Footnotes

^1^ The abbreviations used are:

Mf: microfilariae
AF: adult female
AM: adult male
vL3: vector-derived third-stage infective larvae
iuMf: intrauterine microfilariae
NF: nodule fluid
AEX: anion exchange
BR: Britton-Robinson
ESP: excretory-secretory products
WBE: whole-body extracts
Prx: peroxiredoxin
TGF: transforming growth factor
GST: glutathione transferase
MPP: mitochondrial processing peptidase
TrxR: thioredoxin reductase
PAG: protein ambiguity group
PAL: peptidoglycan-associated lipoprotein
PGLYRP1: peptidoglycan recognition protein
WSP: *Wolbachia* surface protein
*w*Oo: *Wolbachia* endosymbiont of *Onchocerca ochengi.*
