# Supplementary material for: Stage-specific Proteomes from Onchocerca ochengi, Sister Species of the Human River Blindness Parasite, Uncover Adaptations to a Nodular Lifestyle
Source: Mol Cell Proteomics. 2016 May 25;15(8):2554–75. doi: 10.1074/mcp.M115.055640 (PMC4974336; doi:10.1074/mcp.M115.055640)
Supplement: Supplemental Data [file supp_15_8_2554__index.html]

Stage-specific proteomes from Onchocerca ochengi, sister species of the human river blindness parasite, uncover adaptations to a nodular lifestyle — Stage-specific Proteomes from Onchocerca ochengi, Sister Species of the Human River Blindness Parasite, Uncover Adaptations to a Nodular Lifestyle — Stage-specific Proteomes from Onchocerca ochengi — Supplemental Data 

# Stage-specific Proteomes from *Onchocerca ochengi*, Sister Species of the Human River Blindness Parasite, Uncover Adaptations to a Nodular Lifestyle

## Supplemental Data

- Main supplement (.pdf, 2.3 MB) - Main supplement
- Supplemental table S1 (.xlsx, 5.7 MB) - Protein identifications overview.
- Supplemental table S2 (.xlsx, 268 KB) - Extended information for Fig. 2.
- Supplemental table S3 (.xlsx, 688 KB) - Extended information for Fig. 3.
- Supplemental table S4 (.xlsx, 13 KB) - Extended information for Fig. 5.
- Supplemental table S5 (.xlsx, 577 KB) - Extended information for Fig. S7.
