# Supplementary material for: Stage-specific Proteomes from Onchocerca ochengi, Sister Species of the Human River Blindness Parasite, Uncover Adaptations to a Nodular Lifestyle
Source: Mol Cell Proteomics. 2016 May 25;15(8):2554–75. doi: 10.1074/mcp.M115.055640 (PMC4974336; doi:10.1074/mcp.M115.055640)
Supplement: Supplemental Data [file 10.1074_M115.055640_mcp.M115.055640-1.pdf]

Table of contents

Supplemental Fig. S1: Classification of proteins identified from *w*Oo by Clusters of Orthologous Groups………………………………………………………… p. 2

Supplemental Fig. S2: Pfam enrichment analysis for *O. ochengi* compared with published data for *B. malayi*………………………………………………………… p. 3

Supplemental Fig. S3: Distribution of observed immunoglobulin-domain proteins across the *O. ochengi* lifecycle………………………………………………………… p. 4

Extended narrative – results and discussion………………………………………………………… p. 5

Supplemental Fig. S4: Distribution of observed galectins (Pfam PF00337) across the *O. ochengi* lifecycle………………………………………………………… p. 7

Supplemental Fig. S5: Distribution of protein domains involved in intracellular transport across the *O. ochengi* lifecycle………………………………………………………… p. 8

Supplemental Fig. S6: Distribution of enzymes significantly enriched in vL3 across the *O. ochengi* lifecycle………………………………………………………… p. 9

Supplemental Fig. S7: Heat-map of protein abundance across the *O. ochengi* lifecycle………… p. 14

Supplemental Fig. S8: Comparison of the adult secretomes of four filarial species ………………. p. 15

Supplemental Fig. S9: Domain structure of *C. elegans* DIG-1 and its orthologs in filarial nematodes………………………………………………………… p. 16

Supplemental references………………………………………………………… p. 18

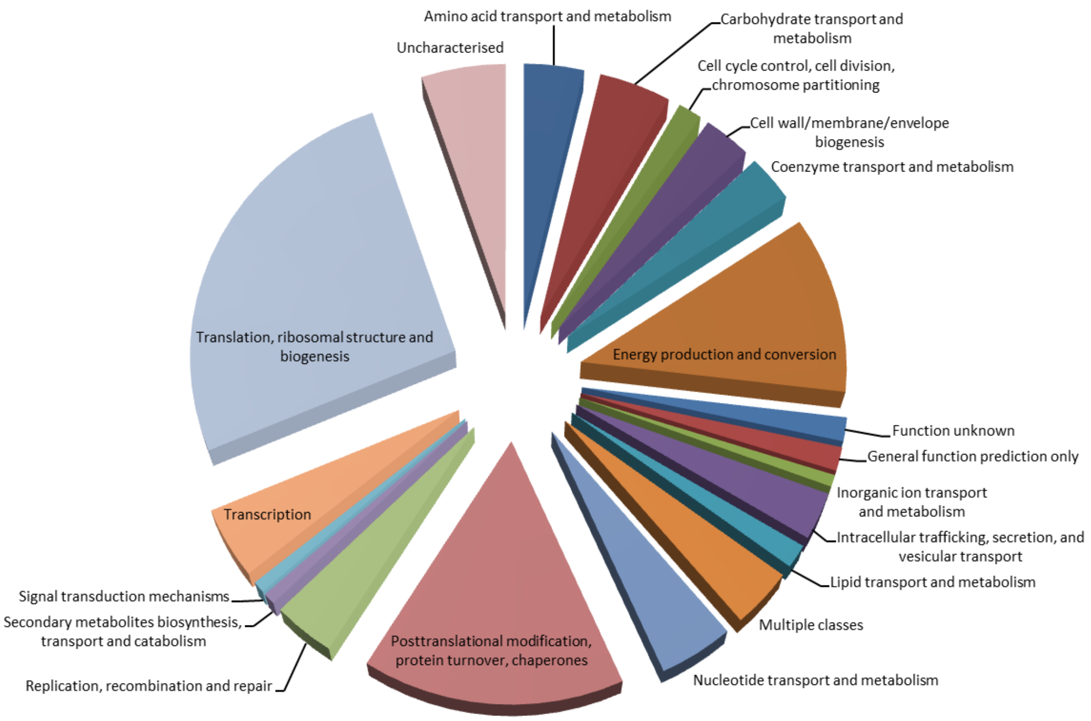

SUPPLEMENTAL FIG. S1. **Classification of proteins identified from *w*Oo by Clusters of Orthologous Groups.** *Wolbachia* proteins from all lifecycle stages combined were categorised (*n* = 135).

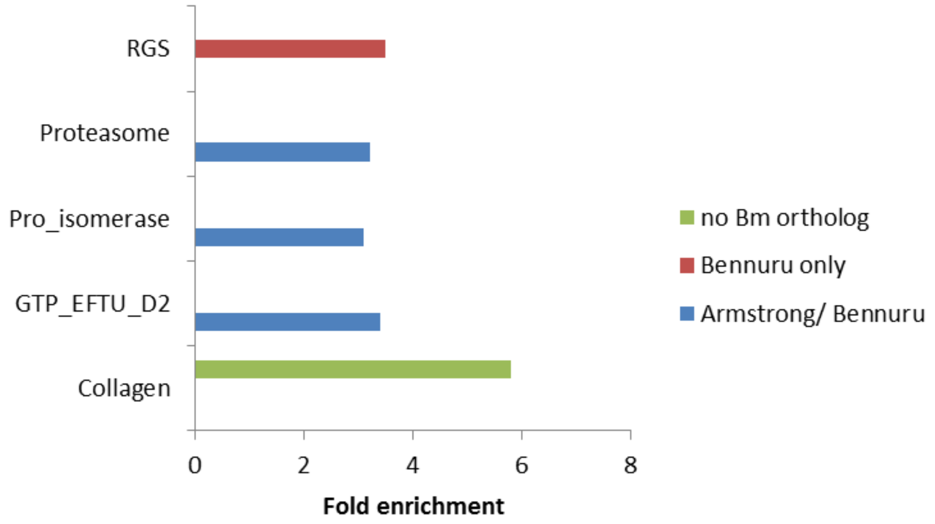

SUPPLEMENTAL FIG. S2. **Pfam enrichment analysis for *O. ochengi* compared with published data for *B. malayi.*** Protein domains that are significantly enriched in the observed proteomes relative to the complete theoretical proteomes are shown for *B. malayi* (Bm, "Bennuru") and *O. ochengi* ("Armstrong"). The enrichment of collagens is specific to *O. ochengi,* as the genes involved lack orthologs in *B. malayi*.

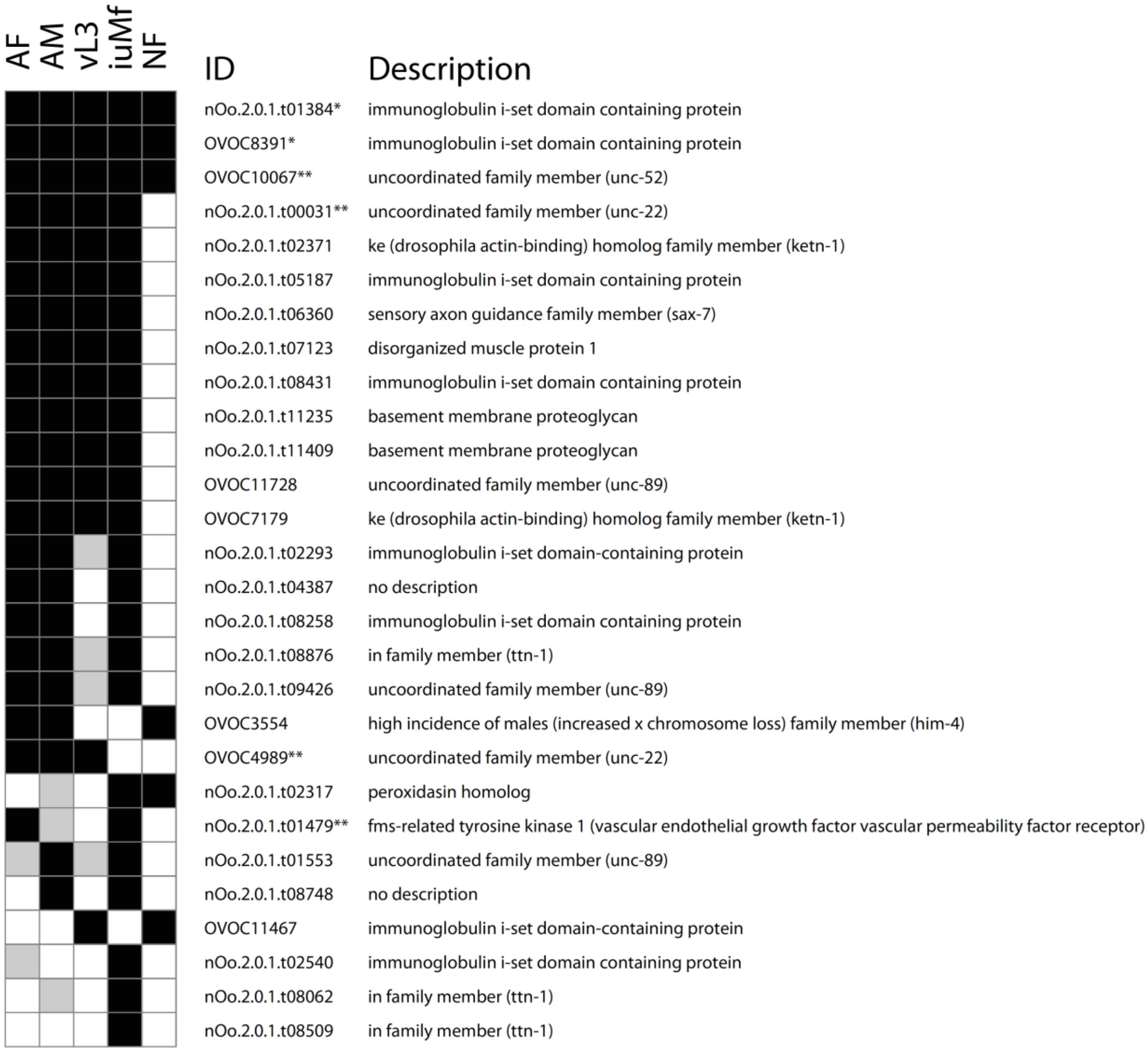

SUPPLEMENTAL FIG. S3. **Distribution of observed immunoglobulin-domain proteins across the *O. ochengi* lifecycle.** Black, detected with ≥2 unique peptides; grey, detected with one unique peptide only; white, not detected. ***Proteins containing Ig domains (Pfam PF00047) only, **proteins containing Ig and I-set (Pfam PF07679) domains (unmarked proteins contain I-set domains only).

Extended narrative – results and discussion

Immunoglobulin domain proteins

Several I-set domain proteins were robustly identified (≥2 peptides) only in iuMf, including a homolog (nOo_02540) of ZIG-1 and two homologs of the C-terminal moiety of titin (supplemental Fig. S3). In *C. elegans*, several products of the *zig* gene family are expressed in the PVT interneuron of the pre-anal ganglion, and are essential for maintenance of correct axon positioning in the ventral nerve cord specifically at the L1 stage (1). The functions of titin in *C. elegans* are more complex, since some isoforms link the dense body of the I-band to the A-band in body wall muscle sarcomeres (2), whereas others bind to lamins in the nuclear envelope and co-ordinate nuclear organisation during interphase (3). However, since titin cannot be detected in the muscle of *C. elegans* L1 (2), the *O. ochengi* homologs are perhaps more likely to be nuclear isoforms with a role in the maturation of Mf.

A single I-set domain protein with homology to *C. elegans* VER-3 (nOo_01479) was observed in AF and iuMf, which is a member of the "vascular endothelial growth factor receptor related" family. The expression of this protein is restricted to the ALA neuron in the dorsal ganglion of the head and the pharyngeal saucer and anal sphincter muscles of *C. elegans*, suggesting a role in neuronal guidance during morphogenesis (4). Surprisingly, two I-set domain proteins were robustly identified only in AM and iuMf, comprising a homolog of UNC-89 and a member of the neuronal cell adhesion molecule family. Whereas UNC-89 is involved in assembly of thick filaments in muscle (5), neuronal cell adhesion molecules are expressed on neurons and glia where they organise cell-to-cell contacts during the formation of neural networks (6). Finally, an I-set domain protein homologous to *C. elegans* DIM-1 was unique in being observed only in vL3 and NF. This protein maintains a strong connection between the myofilament lattice and the muscle cell membrane in *C. elegans* (7), but DIM-1 homologs have also been identified as powerful immunogens from the infective L3 of gastrointestinal parasites such as *Ascaris suum* (8) and *Trichostrongylus colubriformis* (9).

Intracellular transport

A striking feature of the AF and iuMf proteomes was the significantly enrichment of domains associated with intracellular trafficking of vesicles, including Arf and "Adaptin_N" in AF; and Arf, Snf7 and WD40 in iuMf (Fig. 4, supplemental Fig. S5). ADP-ribosylation factor (Arf)-like proteins identified in iuMf included homologs of ARL-1 (also robustly detected in AF) and ARL-3 from *C. elegans.* When expression of these small GTPases was inhibited by RNAi, *C. elegans* embryos arrested at an early stage of development in the case of ARL-1, and at the 2-fold stage of elongation when ARL-3 was targeted (10). Moreover, adult hermaphrodites subjected to RNAi targeting either ARL-1 or ARL-3 were sterilised within 24 hr (10). Subsequent studies have demonstrated that ARL-1 is essential for morphological transitions of the ER during early embryogenesis (11), and both ARL proteins are required

for the production of caveolin-1 bodies that fuse with the oocyte plasma membrane following ovulation and fertilization, which may be a mechanism to prevent polyspermy (12). The “Adaptin_N” domains enriched in AF were located in three subunits from adaptor-related protein complexes (AP)-1 and AP-2, and two subunits from coatomer protein (supplemental Fig. S5). The AP complexes mediate the binding of clathrin-coated vesicles to membranes: AP-1 is involved in transport between the *trans*-Golgi network and endosomes, whereas AP-2 is responsible for traffic between the plasma membrane and early endosomes (13). Conversely, coatomer forms the coat of COP-I vesicles that co-ordinate retrograde transport from the Golgi back to the ER, and is dependent on Arf GTPases for membrane recruitment (14). Both AP-1 and AP-2 are key players in the regulation of Notch signalling during the binary fate decisions that underpin neurogenesis (15), and are also required for secretion of a fibroblast growth factor homolog, EGL-17, which directs the positioning of sex myoblasts in the developing vulva of *C. elegans* (16).

The Snf7 domain proteins that were significantly overrepresented only in iuMf included three charged multivesicular body protein (CHMP) members from the endosomal sorting complex required for transport (supplemental Fig. S5). Although these have cytoplasmic roles in the biogenesis of late endosomes and especially membrane scission (17), we identified a homolog of CHMP-1, which functions secondarily within the nuclear Polycomb group to silence genes epigenetically (18). Moreover, a WD40-domain protein from this iuMf-enriched group, Sec13 (supplemental Fig. S5), also has a dual cytoplasmic and nuclear role. This protein is found both in COP-II vesicles (associated with ER) and in the nuclear pore complex, where it localises to the kinetochores during mitosis (19). Taken together, these observations suggest that intracellular vesicular transport and nuclear gene regulation are key processes during oogenesis, embryogenesis and the maturation of iuMf.

Galectins

In *O. volvulus*, both *Ov*-GBP-1 and *Ov*-GBP-2 are expressed throughout the parasite lifecycle (including in iuMf), although unlike *Ov*-GBP-2, *Ov*-GBP-1 was also detected in eggshells, uterine tissues and pseudocoelomic fluid within AF (20). Furthermore, ESP of immature Mf from *L. sigmodontis* have been shown to contain orthologs of *Ov*-GBP-1 and *Ov*-GBP-2 (21). In the current study, only the *Ov*-GBP-2 ortholog, which unlike *Ov*-GBP-1 contains a signal peptide (20), was detected in NF (supplemental Fig. S4). Notably, two additional *Ov*-GBP-1-like galectins (nOo_08204 and nOo_10621) were identified in all WBE except vL3 (supplemental Fig. S4). High levels of IgE reactivity to galectins have been reported both in onchocerciasis patients and in humans exposed to the dog heartworm *D. immitis*, suggesting a role in allergic-type immunopathology (22, 23). Moreover, galectins in *D. immitis* ESP and expressed on the adult parasite surface have been implicated in fibrin clot lysis via tissue plasminogen activator-dependent induction of the host fibrinolytic system (24, 25).

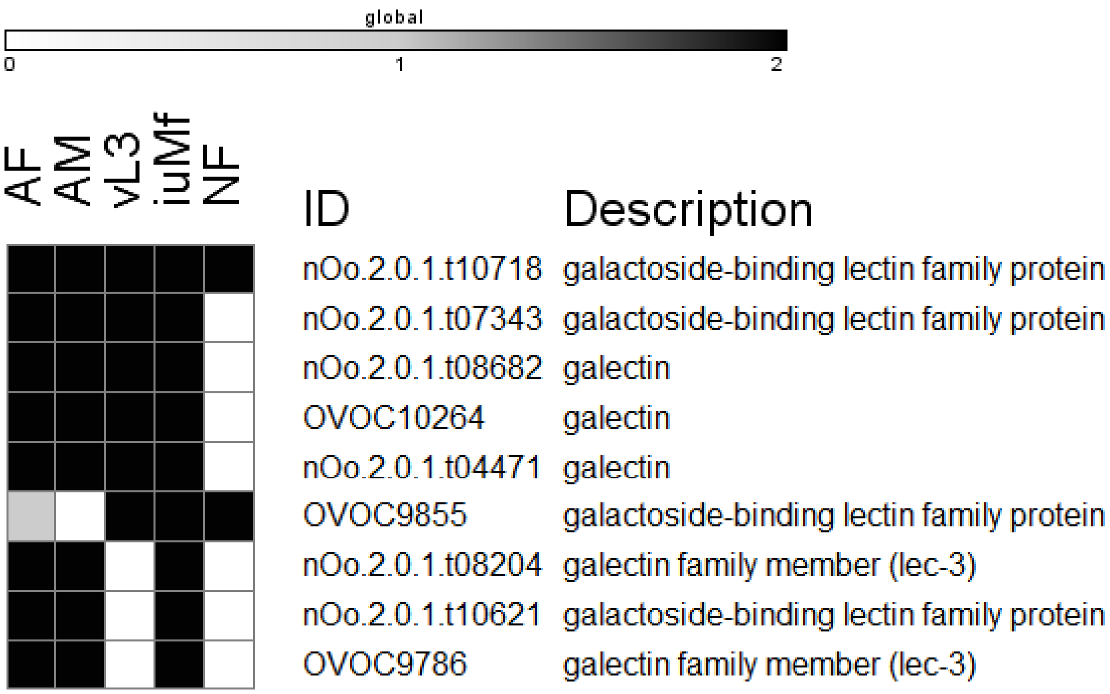

SUPPLEMENTAL FIG. S4. **Distribution of observed galectins (Pfam PF00337) across the *O. ochengi* lifecycle.** Black, detected with ≥2 unique peptides; grey, detected with one unique peptide only; white, not detected.

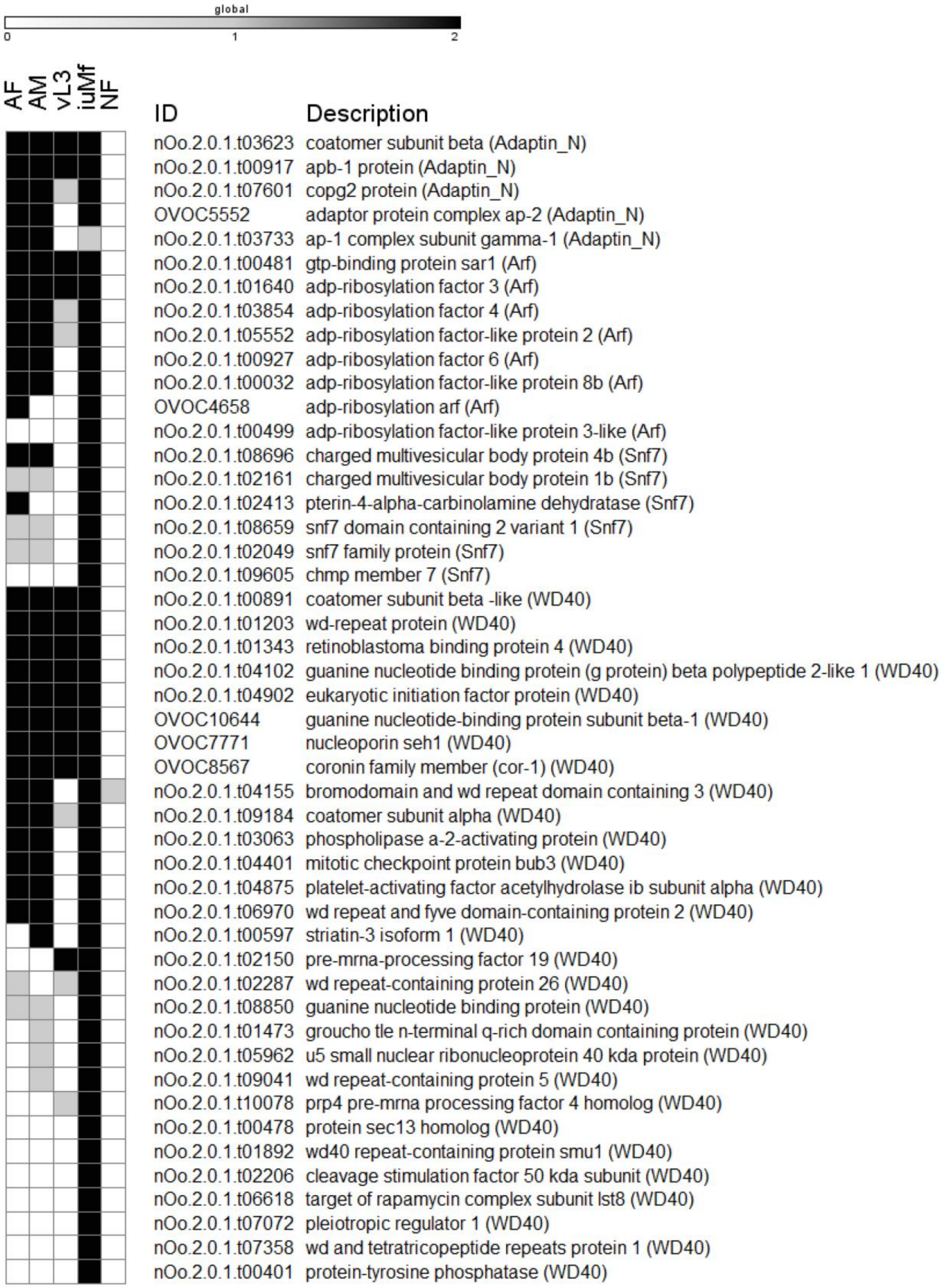

SUPPLEMENTAL FIG. S5. **Distribution of protein domains involved in intracellular transport across the *O. ochengi* lifecycle.** Adaptin_N (Pfam PF01602); Arf (Pfam PF00025); Snf7 (Pfam PF03357); WD40 (Pfam PF00400). Black, detected with ≥2 unique peptides; grey, detected with one unique peptide only; white, not detected.

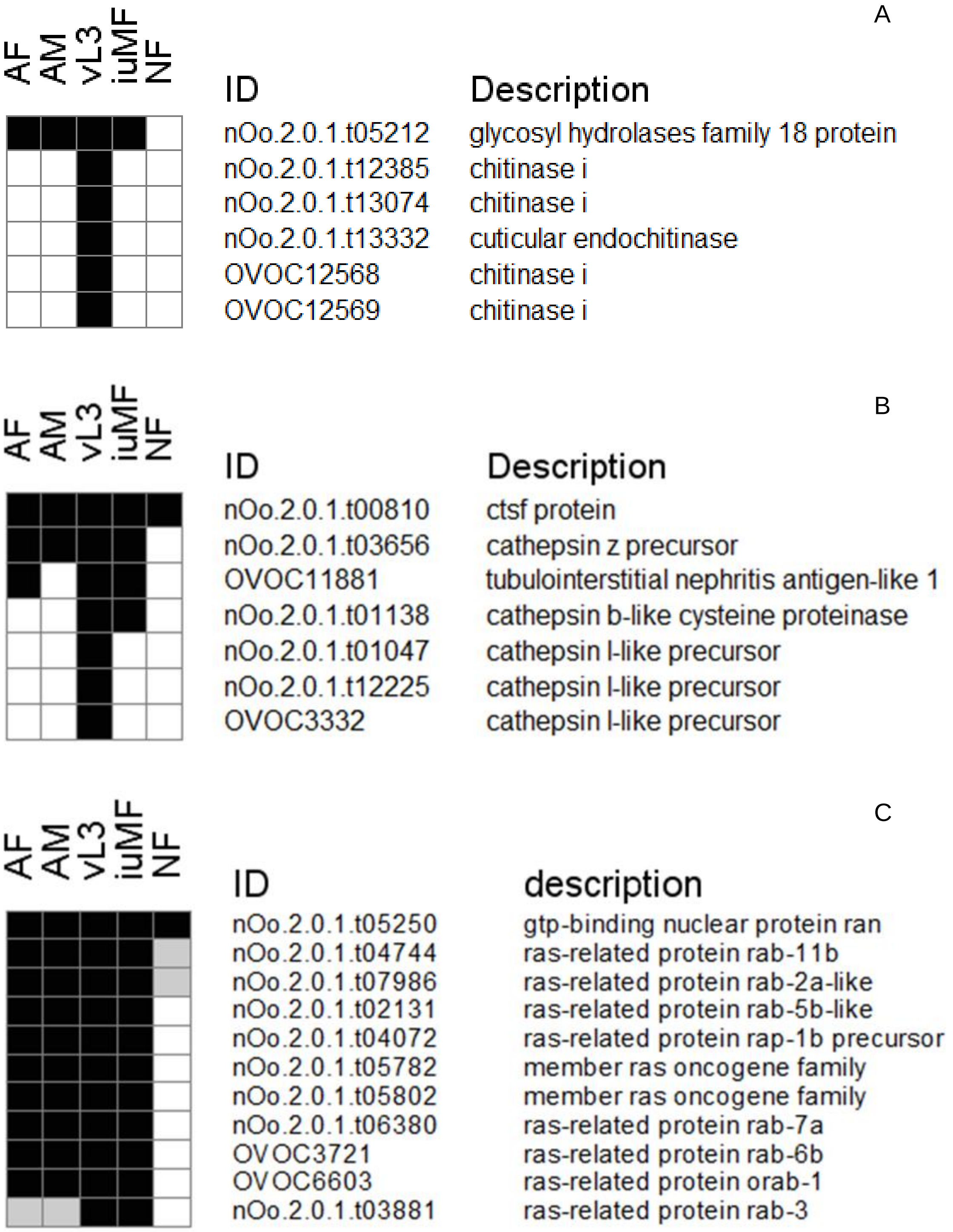

SUPPLEMENTAL FIG. S6. **Distribution of enzymes significantly enriched in vL3 across the *O. ochengi* lifecycle.** (*A*) Chitinases (Pfam PF00704), (*B*) Cathepsin-like proteases (Pfam PF00112) and (*C*) Ras small GTPases (Pfam PF00071). Black, detected with ≥2 unique peptides; grey, detected with one unique peptide only; white, not detected.

Enzymes specifically enriched in vL3

Filarial chitinases were first identified from transcripts that were found to be highly expressed in L3 cDNA libraries from the rodent filaria *Acanthocheilonema viteae* (26). Moreover, in *O. volvulus, Ov*-CHI-1 was localised exclusively within secretory granules of the glandular oesophagus in L3 (27). As revealed by RNAi experiments in *A. viteae*, chitinases are required for moulting of L3 (although chitin is not present in nematode cuticles) and hatching of Mf, and they may have other roles in the physiology of AF (28) and during larval development in the vector (27). In the current study, we identified a single chitinase (nOo_05212) in all WBE (supplemental Fig. S6*A*) with a predicted molecular weight of 42 kDa; this corresponds to the size of a second *O. volvulus* chitinase reported by Wu *et al*. (27) in adult worms of both sexes, as well as L3. The other five chitinases we detected were strictly vL3-specific (supplemental Fig. S6*A*), although some lacked full-length chitinase domains and probably represent split gene models. The anthelminthic closantel and a group of natural compounds, β-carboline alkaloids, were recently demonstrated to inhibit the activity of *Ov*-CHI-1 *in vitro*, which led to significant inhibition of the L3 – L4 moult (29, 30).

The second large enzyme family that was enriched in vL3 was the cathepsin-like proteases. Of these, only three cathepsin-L-like precursors were restricted to vL3 extracts (supplemental Fig. S6*B*), which corresponds with the high expression of cathepsin-L-like cysteine proteases in the L3 of both *O. volvulus* and *Brugia pahangi*, and their essential role in moulting (31, 32). We also identified a cathepsin-F-like protease in all WBE and NF that was orthologous to a *B. malayi* transcript reported by Guiliano *et al*. (31); and a cathepsin-Z-like protease from all WBE that was 99% identical to that identified in *O. volvulus* (33) (supplemental Fig. S6*B*). Finally, a cathepsin-B-like protease was detected in iuMf and vL3 only (supplemental Fig. S6*B*). In addition to their role in moulting, cathepsin-Z-like proteases are expressed in the hypodermis, pharynx and gonads of adult *C. elegans*, and disruption of the gene leads to partial embryonic lethality and morphological defects at later stages (34).

The final domain specifically enriched in vL3 was the Ras superfamily of small GTPases. In the current study, most Ras proteins were detected in all WBE, but a homolog of *C. elegans* Rab3 (nOo_03881) was robustly identified in vL3 and iuMf only (supplemental Fig. S6*C*). This Ras protein is required for regulation of synaptic vesicle recruitment in the nervous system of the pharynx and is not expressed exclusively in larvae (35). However, other Ras superfamily members are specifically expressed during embryogenesis and larval development in *C. elegans*, and have roles in morphogenesis and axon path-finding (36, 37). Thus, the functions of Ras superfamily members such as nOo_03881 deserve to be explored during filarial development.

Quantitative differences between stages

In addition to the chitinases and cathepsins already highlighted as enriched in L3, a cuticlin homolog previously identified in post-infective L3 and L4 cDNA libraries from *B. pahangi* (38) was one of the more abundant vL3-specific proteins (supplemental Table S5). However, as expected, the dominant protein found only in vL3 was abundant larval

transcript protein-1, the key immunomodulatory protein secreted by L3 during establishment of infection (39, 40). Other immunomodulators quantified in vL3 included two isoforms of activation-associated secreted proteins (ASPs), for which ASP-1 was more abundant than ASP-2 (supplemental Fig. 7*A*). Members of the ASP family have angiogenic properties (41) and stimulate Th-1-biased cellular responses, which has led to development of *Ov*-ASP-1 as an adjuvant for viral vaccines (42).

Preparations from iuMf exhibited the greatest number of stage-specific proteins, reflecting their greater proteomic diversity overall. By far the largest group in this stage were RNA-binding proteins such as splicing factors (supplemental Fig. 7*A*; supplemental Table S5), suggesting that the incomplete developmental status of iuMf is characterised by heightened activity in the spliceosome. The most abundant unique protein in iuMf was an enzyme involved in pyrimidine synthesis (uridine phosphorylase), although mitochondrial proteins such as malate dehydrogenase, hexokinase type II, L-2-hydroxyglutarate dehydrogenase, and apoptosis-inducing factor-1 were also well represented. Interestingly, a fatty acid retinoid-binding protein precursor orthologous to *Ov*-FAR-2 was very abundant in the iuMf-specific dataset, even though *Ov*-FAR-2 was identified from *O. volvulus* L3 cDNA libraries (43). This protein is clearly distinct from Oo-FAR-1 (52% coverage, 25% identity), an antigen expressed in all lifecycle stages which was evaluated in a vaccine trial against *O. ochengi* (44).

Sex-specific gene and protein expression have been explored in *B. malayi*, and in agreement with an expression array study (45), we found protein serine-threonine phosphatases (PSPs) to be enriched in AM in both the Pfam ("Metallophos", Fig. 4) and the Hi-3 analyses (supplemental Fig. 7*A*). Alongside the major sperm proteins (the most abundant unique proteins in AM; supplemental Fig. 7*A*; supplemental Table S5), PSPs are among the most distinctive male-specific genes in nematodes, which have been associated with spermatogenesis in *C. elegans* (46), *Haemonchus contortus* (47), *Ascaris suum* (48), *Trichostrongylus vitrinus* (49) and *Toxocara canis* (50). In *C. elegans*, mutation of one PSP family member, calcineurin, leads to aberrant sperm morphology and a reduction in quantity (46), whereas RNAi of another PSP group (Glc-seven phosphatases) results in defective sperm chromosome segregation (51). Other male-specific filarial proteins reported previously that are corroborated in the current study include PDZ-domain proteins [associated with AM in *B. malayi* (52)] and adenylate kinase isoenzyme-1, which is considered a male-enriched protein in *B. malayi* (52) and is also the dominant surface protein on AM in *L. sigmodontis* (21). Furthermore, we noted that the Pfam and Hi-3 datasets concurred in the overrepresentation of malic enzyme in AM (Fig. 4 and supplemental Fig. 7*A*). Adult *Onchocerca* spp. are homolactate fermenters, and the presence of cytosolic NADP-dependent malic enzymes in several species has been reported previously from mixed-sex adult worm extracts (53, 54).These enzymes are a key inhibitory target of the adulticidal drug, suramin (53), although to the best of our knowledge, potential differences in malic enzyme activity between AF and AM of *Onchocerca* spp. have not been investigated previously. Finally, AM showed a remarkable diversity of uncharacterised

proteins lacking any conserved domains, several of which were highly abundant (supplemental Fig. 7*A*; supplemental Table S5).

In contrast with AM, AF exhibited a marked paucity of unique proteins (six in total; supplemental Fig. 7*A*; supplemental Table S5). Although this is probably due largely to the overlap between the AF and iuMf proteomes, the implication is that AM and AF display very similar protein expression patterns outside the reproductive tract. The most abundant stage-specific protein in AF was a cuticular collagen, although cystathionine beta-synthase was also well represented. Notably, RNAi experiments have demonstrated that this enzyme is highly expressed in murine granulosa cells and is required for oocyte maturation (55). Two other unusual proteins were uniquely abundant in AF: a homolog of a mitochondrial porin in *C. elegans*, TOMM-40, and a bactericidal permeability-increasing superfamily domain protein (supplemental Fig. 7*A*). TOMM-40 forms a hydrophilic pore in the mitochondrial translocase and is required for importation of nuclear-encoded preproteins into the organelle (56). This porin is essential for embryogenesis and larval development, since inactivation of TOMM-40 causes lack of responsiveness to the insulin-like peptide DAF-28 and larval arrest in *C. elegans* (57). The bactericidal permeability-increasing superfamily domain protein is more enigmatic, but another nematode protein containing this domain is NRF-5 in *C. elegans*, which is secreted from the intestine and may be involved in drug detection and transport, in addition to trafficking of yolk proteins to oocytes (58). Moreover, OBI-1 in *Pristionchus pacificus* harbours the same domain and is required for neutralisation of a sex pheromone secreted by its beetle host, which can otherwise induce arrest of embryogenesis and inhibition of exit from the dauer (dormant) larval stage (59). Intriguingly, *P. pacificus* OBI-1 is expressed in various tissues of the vulva as well as in the amphid neuron sheath. Taken together, these observations suggest that previously unexplored AF-enriched proteins may be essential for partitioning of resources during embryogenesis and perhaps host-parasite crosstalk or sexual communication.

DIG-1 in nodule fluid

The etymology of "DIG-1" is derived from the phenotype of *C. elegans* mutants, which have a displaced gonad and associated defects in the positioning of HSN neurons that innervate the uterine and vulval musculature (60), as well as a twisted pharynx (61). Unlike its homolog in *O. ochengi* (nOo_01384), *C. elegans* DIG-1 has a classical signal peptide and a predicted molecular weight of ~1.37 MDa, rendering it the largest secreted protein described to date (62) (supplemental Fig. S9). It has roles during embryogenesis in the normal fasciculation of head sensory neurons (62) and ensheaths ganglia and fascicles in the ventral nerve cord and head region of adults (63). The presence of the DIG-1 homolog (and other basement membrane proteins involved in neuron development) within the nodule fluid is surprising. These proteins may simply be expressed at high levels during embryogenesis and released during parturition, or they could have evolved to incorporate entirely different roles in the context of parasitism, such as immunomodulation or establishment of a permissive environment for the adult worms. Whatever their function,

orthologs of nOo_01384 have been reported from the ESP of both *B. malayi* (64, 65) and *L. sigmodontis* (21), and orthologs are also present in the genomes of *D. immitis* and *O. volvulus*. Notably, most of the filarial orthologs are approximately half the size of *C. elegans* DIG-1, with much reduced six-bladed β-propeller domains in the central region and an absence of a signal peptide, but full retention of immunoglobulin-fold and von Willebrand factor type-A domains in the N- and C-terminal portions, respectively (supplemental Fig. S9). However, we detected a unique peptide from the C-terminal portion of the *O. volvulus* ortholog (OVOC8391) which was not present in nOo_01384, suggesting that the multitude of intron-exon boundaries has led to errors in gene models. Nevertheless, the *L. sigmodontis* ortholog appeared to be distinct from the other filarial DIG-1-like proteins in the extent of its diminution, with a predicted molecular weight of only ~226 kDa, and a complete absence of β-propeller and von Willebrand factor type-A domains (supplemental Fig. S9). Interestingly, this ortholog did retain a signal peptide (supplemental Fig. S9).

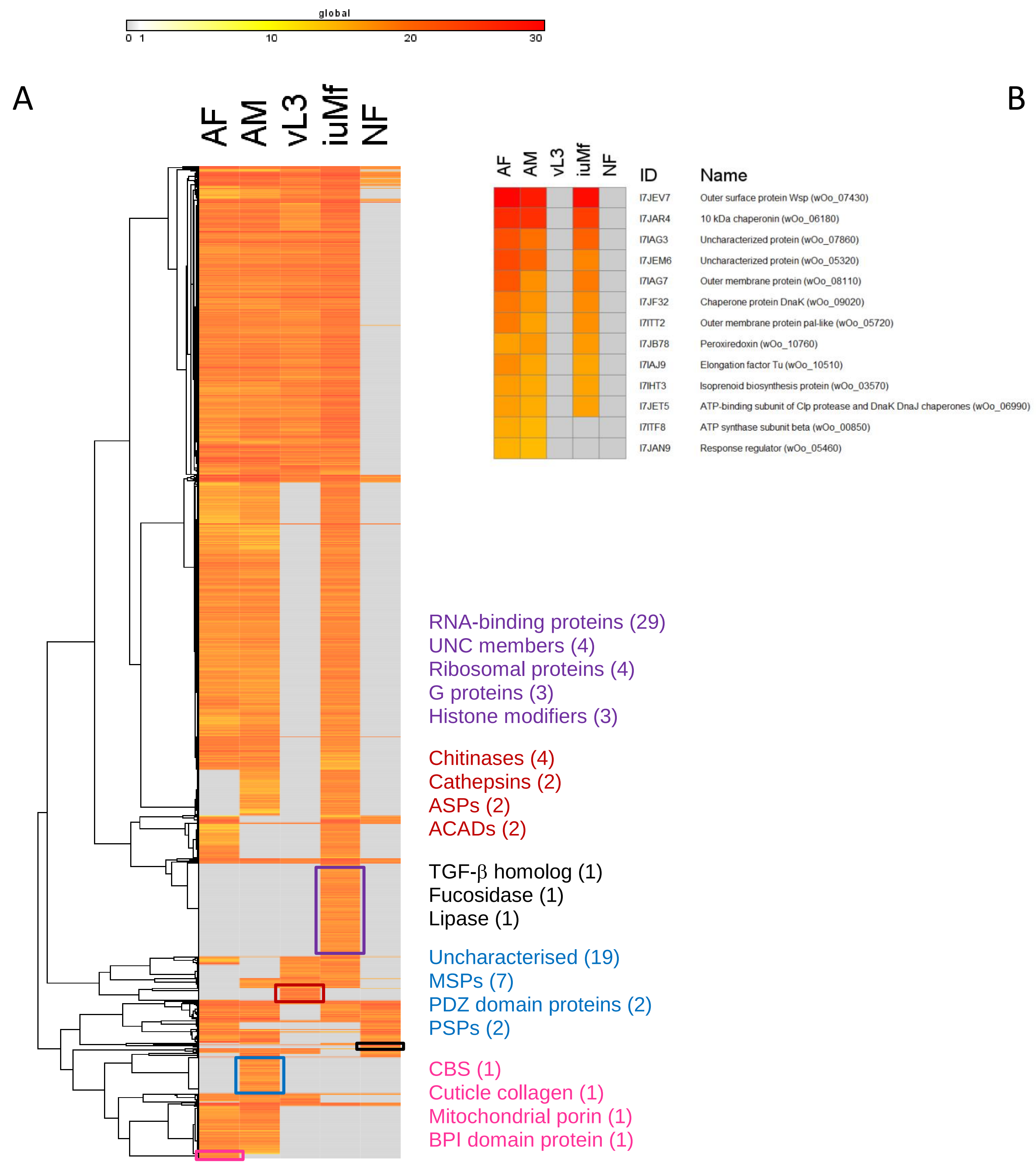

SUPPLEMENTAL FIG. S7. **Heat-map of protein abundance across the *O. ochengi* lifecycle.** Proteins were quantified by the Hi-3 method for (*A*) *O. ochengi* and (*B*) *w*Oo, and dendrograms were generated by hierarchical clustering based on pair-wise distance. Stage-specific clusters of expression and selected proteins of interest are highlighted for iuMf (purple), vL3 (red), NF (black), AM (blue) and AF (pink). UNC, uncoordinated; G protein, guanine nucleotide-binding protein; ASP, activation-associated secreted proteins; ACAD, acyl-CoA dehydrogenase; TGF, transforming growth factor; MSP, major sperm protein; PSP, protein serine-threonine phosphatase; CBS, cystathionine beta-synthase; BPI, bactericidal permeability-increasing. Complete lists of proteins identified in each cluster are provided in supplemental Table S5.

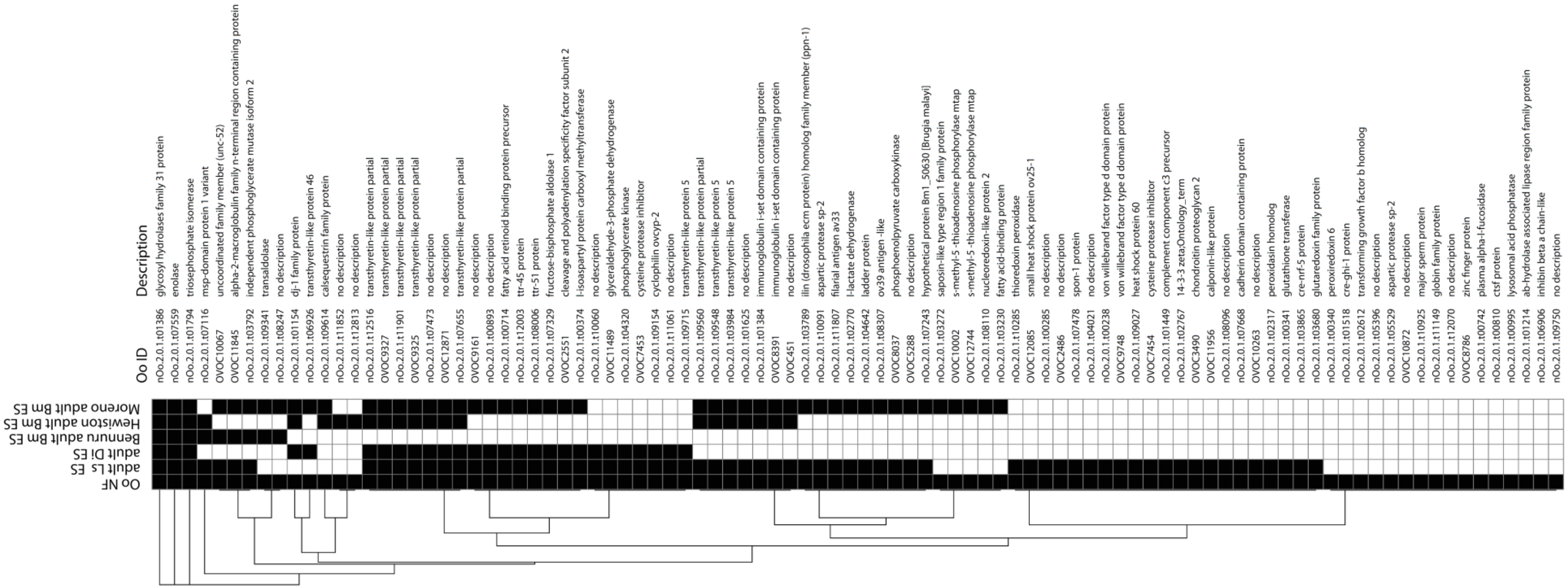

SUPPLEMENTAL FIG. S8. **Comparison of the adult secretomes of four filarial species.** Orthologs of *O. ochengi* proteins detected in the NF secretome were mapped to published identifications for *B. malayi* (64-66), *L. sigmodontis* (21) and *D. immitis* (67). Dendrograms were generated by hierarchical clustering based on pair-wise distance. Note that only orthologs of proteins detected in NF are shown for the other three species.

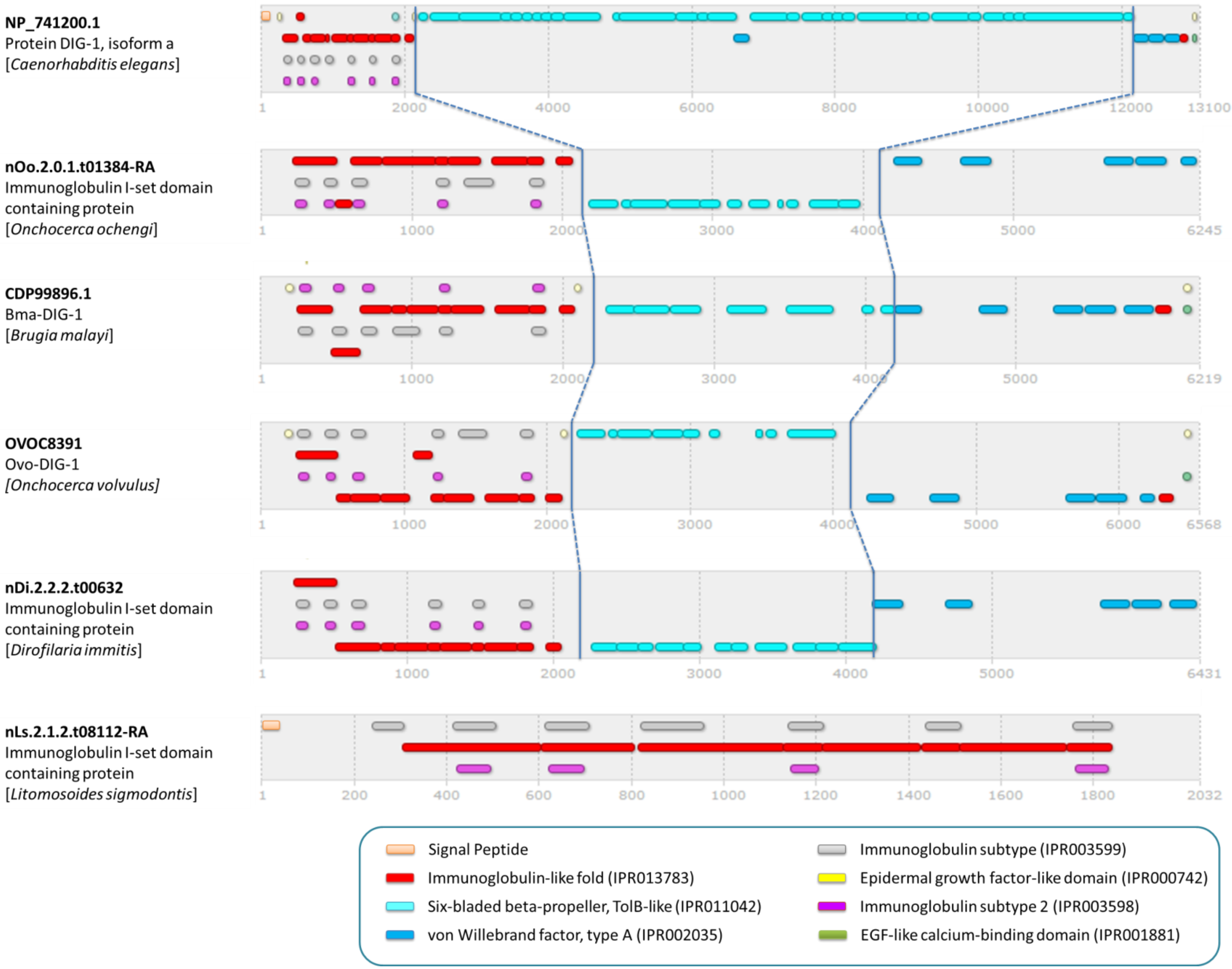
NP_741200.1
Protein DIG-1, isoform a
[Caenorhabditis elegans]
1 2000 4000 6000 8000 10000 12000 13100
nOo.2.0.1.t01384-RA
Immunoglobulin I-set domain containing protein
[Onchocerca ochengi]
1 1000 2000 3000 4000 5000 6245
CDP99896.1
Bma-DIG-1
[Brugia malayi]
1 1000 2000 3000 4000 5000 6219
OVOC8391
Ovo-DIG-1
[Onchocerca volvulus]
1 1000 2000 3000 4000 5000 6000 6568
nDi.2.2.2.t00632
Immunoglobulin I-set domain containing protein
[Dirofilaria immitis]
1 1000 2000 3000 4000 5000 6431
nLs.2.1.2.t08112-RA
Immunoglobulin I-set domain containing protein
[Litomosoides sigmodontis]
1 200 400 600 800 1000 1200 1400 1600 1800 2032
Signal Peptide
Immunoglobulin-like fold (IPR013783)
Six-bladed beta-propeller, TolB-like (IPR011042)
von Willebrand factor, type A (IPR002035)
Immunoglobulin subtype (IPR003599)
Epidermal growth factor-like domain (IPR000742)
Immunoglobulin subtype 2 (IPR003598)
EGF-like calcium-binding domain (IPR001881)

SUPPLEMENTAL FIG. S9. **Domain structure of *C. elegans* DIG-1 and its orthologs in filarial nematodes.** The protein domain architecture for DIG-1 from *C. elegans* is compared with that in *O. ochengi, B. malayi, O. volvulus, D. immitis* and *L. sigmodontis*. Vertical lines highlight the reduction of the six-bladed β-propeller domain in filarial proteins.

SUPPLEMENTAL REFERENCES

1. Aurelio, O., Hall, D. H., and Hobert, O. (2002) Immunoglobulin-domain proteins required for maintenance of ventral nerve cord organization. *Science* 295, 686-690

2. Flaherty, D. B., Gernert, K. M., Shmeleva, N., Tang, X. X., Mercer, K. B., Borodovsky, M., and Benian, G. M. (2002) Titins in *C-elegans* with unusual features: Coiled-coil domains, novel regulation of kinase activity and two new possible elastic regions. *J. Mol. Biol.* 323, 533-549

3. Zastrow, M. S., Flaherty, D. B., Benian, G. M., and Wilson, K. L. (2006) Nuclear titin interacts with A- and B-type lamins in vitro and in vivo. *J. Cell Sci.* 119, 239-249

4. Popovici, C., Isnardon, D., Birnbaum, D., and Roubin, R. (2002) *Caenorhabditis elegans* receptors related to mammalian vascular endothelial growth factor receptors are expressed in neural cells. *Neurosci. Lett.* 329, 116-120

5. Benian, G. M., Tinley, T. L., Tang, X., and Borodovsky, M. (1996) The *Caenorhabditis elegans* gene *unc-89*, required fpr muscle M-line assembly, encodes a giant modular protein composed of Ig and signal transduction domains. *J. Cell Biol.* 132, 835-848

6. Katidou, M., Vidaki, M., Strigini, M., and Karagogeos, D. (2008) The immunoglobulin family of neuronal cell adhesion molecules: Lessons from animal models and correlation with human disease. *Biotech. J.* 3, 1564-1580

7. Rogalski, T. M., Gilbert, M. M., Devenport, D., Norman, K. R., and Moerman, D. G. (2003) DIM-1, a novel imnunoglobulin superfamily protein in *Caenorhabditis elegans*, is necessary for maintaining bodywall muscle integrity. *Genetics* 163, 905-915

8. Tsuji, N., Kasuga-Aoki, H., Isobe, T., Arakawa, T., and Matsumoto, Y. (2002) Cloning and characterisation of a highly immunoreactive 37 kDa antigen with multi-immunoglobulin domains from the swine roundworm *Ascaris suum*. *Int. J. Parasitol.* 32, 1739-1746

9. Kiel, M., Josh, P., Jones, A., Windon, R., Hunt, P., and Kongsuwan, K. (2007) Identification of immuno-reactive proteins from a sheep gastrointestinal nematode, *Trichostrongylus colubriformis*, using two-dimensional electrophoresis and mass spectrometry. *Int. J. Parasitol.* 37, 1419-1429

10. Li, Y., Kelly, W. G., Logsdon, J. M., Jr., Schurko, A. M., Harfe, B. D., Hill-Harfe, K. L., and Kahn, R. A. (2004) Functional genomic analysis of the ADP-ribosylation factor family of GTPases: phylogeny among diverse eukaryotes and function in *C. elegans*. *FASEB J.* 18, 1834-1850

11. Poteryaev, D., Squirrell, J. M., Campbell, J. M., White, J. G., and Spang, A. (2005) Involvement of the actin cytoskeleton and homotypic membrane fusion in ER dynamics in *Caenorhabditis elegans*. *Mol. Biol. Cell* 16, 2139-2153

12. Sato, K., Sato, M., Audhya, A., Oegema, K., Schweinsberg, P., and Grant, B. D. (2006) Dynamic regulation of caveolin-1 trafficking in the germ line and embryo of *Caenorhabditis elegans*. *Mol. Biol. Cell* 17, 3085-3094

13. Owen, D. J., Collins, B. M., and Evans, P. R. (2004) Adaptors for clathrin coats: structure and function. *Annu. Rev. Cell Dev. Biol.* 20, 153-191

14. Eugster, A., Frigerio, G., Dale, M., and Duden, R. (2000) COP I domains required for coatomer integrity, and novel interactions with ARF and ARF-GAP. *EMBO J.* 19, 3905-3917

15. Cotton, M., Benhra, N., and Le, B. R. (2013) Numb inhibits the recycling of Sanpodo in *Drosophila* sensory organ precursor. *Curr. Biol.* 23, 581-587

16. Kamikura, D. M., and Cooper, J. A. (2006) Clathrin interaction and subcellular localization of Ce-DAB-1, an adaptor for protein secretion in *Caenorhabditis elegans*. *Traffic* 7, 324-336

17. Schuh, A. L., Hanna, M., Quinney, K., Wang, L., Sarkeshik, A., Yates, J. R., III, and Audhya, A. (2015) The VPS-20 subunit of the endosomal sorting complex ESCRT-III exhibits an open conformation in the absence of upstream activation. *Biochem. J.* 466, 625-637

18. Stauffer, D. R., Howard, T. L., Nyun, T., and Hollenberg, S. M. (2001) CHMP1 is a novel nuclear matrix protein affecting chromatin structure and cell-cycle progression. *J. Cell Sci.* 114, 2383-2393

19. Loiodice, I., Alves, A., Rabut, G., Van, O. M., Ellenberg, J., Sibarita, J. B., and Doye, V. (2004) The entire Nup107-160 complex, including three new members, is targeted as one entity to kinetochores in mitosis. *Mol. Biol. Cell* 15, 3333-3344

20. Joseph, G. T., Huima, T., Klion, A., and Lustigman, S. (2000) A novel developmentally regulated galectin of *Onchocerca volvulus*. *Mol. Biochem. Parasitol.* 106, 187-195

21. Armstrong, S. D., Babayan, S. A., Lhermitte-Vallarino, N., Gray, N., Xia, D., Martin, C., Kumar, S., Taylor, D. W., Blaxter, M. L., Wastling, J. M., and Makepeace, B. L. (2014) Comparative analysis of the secretome from a model filarial nematode (*Litomosoides sigmodontis*) reveals maximal diversity in gravid female parasites. *Mol. Cell Proteomics* 13, 2527-2544

22. Klion, A. D., and Donelson, J. E. (1994) OvGalBP, a filarial antigen with homology to vertebrate galactoside-binding proteins. *Mol. Biochem. Parasitol.* 65, 305-315

23. Pou-Barreto, C., Quispe-Ricalde, M. A., Morchon, R., Vazquez, C., Genchi, M., Postigo, I., Valladares, B., and Simon, F. (2008) Galectin and aldolase-like molecules are responsible for the specific IgE response in humans exposed to *Dirofilaria immitis*. *Parasite Immunol.* 30, 596-602

24. Gonzalez-Miguel, J., Morchon, R., Mellado, I., Carreton, E., Montoya-Alonso, J. A., and Simon, F. (2012) Excretory/secretory antigens from *Dirofilaria immitis* adult worms interact with the host fibrinolytic system involving the vascular endothelium. *Mol. Biochem. Parasitol.* 181, 134-140

25. Gonzalez-Miguel, J., Morchon, R., Carreton, E., Montoya-Alonso, J. A., and Simon, F. (2013) Surface associated antigens of *Dirofilaria immitis* adult worms activate the host fibrinolytic system. *Vet. Parasitol.* 196, 235-240

26. Adam, R., Kaltmann, B., Rudin, W., Friedrich, T., Marti, T., and Lucius, R. (1996) Identification of chitinase as the immunodominant filarial antigen recognized by sera of vaccinated rodents. *J. Biol. Chem.* 271, 1441-1447

27. Wu, Y., Egerton, G., Underwood, A. P., Sakuda, S., and Bianco, A. E. (2001) Expression and secretion of a larval-specific chitinase (family 18 glycosyl hydrolase) by the infective stages of the parasitic nematode, *Onchocerca volvulus*. *J. Biol. Chem.* 276, 42557-42564

28. Tachu, B., Pillai, S., Lucius, R., and Pogonka, T. (2008) Essential role of chitinase in the development of the filarial nematode *Acanthocheilonema viteae*. *Infect. Immun.* 76, 221-228

29. Gloeckner, C., Garner, A. L., Mersha, F., Oksov, Y., Tricoche, N., Eubanks, L. M., Lustigman, S., Kaufmann, G. F., and Janda, K. D. (2010) Repositioning of an existing drug for the neglected tropical disease onchocerciasis. *Proc. Natl. Acad. Sci. USA* 107, 3424-3429

30. Gooyit, M., Tricoche, N., Javor, S., Lustigman, S., and Janda, K. D. (2015) Exploiting the polypharmacology of beta-carbolines to disrupt *O. volvulus* molting. *ACS Med. Chem. Lett.* 6, 339-343

31. Guiliano, D. B., Hong, X., McKerrow, J. H., Blaxter, M. L., Oksov, Y., Liu, J., Ghedin, E., and Lustigman, S. (2004) A gene family of cathepsin L-like proteases of filarial nematodes are associated with larval molting and cuticle and eggshell remodeling. *Mol. Biochem. Parasitol.* 136, 227-242

32. Lustigman, S., Zhang, J., Liu, J., Oksov, Y., and Hashmi, S. (2004) RNA interference targeting cathepsin L and Z-like cysteine proteases of *Onchocerca volvulus* confirmed their essential function during L3 molting. *Mol. Biochem. Parasitol.* 138, 165-170

33. Lustigman, S., McKerrow, J. H., Shah, K., Lui, J., Huima, T., Hough, M., and Brotman, B. (1996) Cloning of a cysteine protease required for the molting of *Onchocerca volvulus* third stage larvae. *J. Biol. Chem.* 271, 30181-30189

34. Hashmi, S., Zhang, J., Oksov, Y., and Lustigman, S. (2004) The *Caenorhabditis elegans* cathepsin Z-like cysteine protease, Ce-CPZ-1, has a multifunctional role during the worms' development. *J. Biol. Chem.* 279, 6035-6045

35. Nonet, M. L., Staunton, J. E., Kilgard, M. P., Fergestad, T., Hartwieg, E., Horvitz, H. R., Jorgensen, E. M., and Meyer, B. J. (1997) *Caenorhabditis elegans rab-3* mutant synapses exhibit impaired function and are partially depleted of vesicles. *J. Neurosci.* 17, 8061-8073

36. Lundquist, E. A. (2006) Small GTPases, In: Greenwald, I. (ed), WormBook, pp. 1-18

37. Chen, W. N., Lim, H. H., and Lim, L. (1993) The Cdc42 Homolog from *Caenorhabditis elegans* - complementation of yeast mutation. *J. Biol. Chem.* 268, 13280-13285

38. Lewis, E., Hunter, S. J., Tetley, L., Nunes, C. P., Bazzicalupo, P., and Devaney, E. (1999) *cut-1*-like genes are present in the filarial nematodes, *Brugia pahangi* and *Brugia malayi*, and, as in other nematodes, code for components of the cuticle. *Mol. Biochem. Parasitol.* 101, 173-183

39. Wu, Y., Egerton, G., Pappin, D. J., Harrison, R. A., Wilkinson, M. C., Underwood, A., and Bianco, A. E. (2004) The Secreted Larval Acidic Proteins (SLAPs) of *Onchocerca* spp. are encoded by orthologues of the *alt* gene family of *Brugia malayi* and have host protective potential. *Mol. Biochem. Parasitol.* 134, 213-224

40. Gomez-Escobar, N., Bennett, C., Prieto-Lafuente, L., Aebischer, T., Blackburn, C. C., and Maizels, R. M. (2005) Heterologous expression of the filarial nematode *alt* gene products reveals their potential to inhibit immune function. *BMC Biol.* 3, 8

41. Tawe, W., Pearlman, E., Unnasch, T. R., and Lustigman, S. (2000) Angiogenic activity of *Onchocerca volvulus* recombinant proteins similar to vespid venom antigen 5. *Mol. Biochem. Parasitol.* 109, 91-99

42. MacDonald, A. J., Cao, L., He, Y., Zhao, Q., Jiang, S., and Lustigman, S. (2005) rOv-ASP-1, a recombinant secreted protein of the helminth *Onchocerca volvulus*, is a potent adjuvant for inducing antibodies to ovalbumin, HIV-1 polypeptide and SARS-CoV peptide antigens. *Vaccine* 23, 3446-3452

43. Cho-Ngwa, F., Liu, J., and Lustigman, S. (2010) The *Onchocerca volvulus* cysteine proteinase inhibitor, Ov-CPI-2, is a target of protective antibody response that increases with age. *PLoS Negl. Trop. Dis.* 4, e800

44. Makepeace, B. L., Jensen, S. A., Laney, S. J., Nfon, C. K., Njongmeta, L. M., Tanya, V. N., Williams, S. A., Bianco, A. E., and Trees, A. J. (2009) Immunisation with a multivalent, subunit vaccine reduces patent infection in a natural bovine model of onchocerciasis during intense field exposure. *PLoS Negl. Trop. Dis.* 3, e544

45. Li, B. W., Rush, A. C., Jiang, D. J., Mitreva, M., Abubucker, S., and Weil, G. J. (2011) Gender-associated genes in filarial nematodes are important for reproduction and potential intervention targets. *PLoS Negl. Trop. Dis.* 5, e947

46. Bandyopadhyay, J., Lee, J., Lee, J., Lee, J. I., Yu, J. R., Jee, C., Cho, J. H., Jung, S., Lee, M. H., Zannoni, S., Singson, A., Kim, D. H., Koo, H. S., and Ahnn, J. (2002) Calcineurin, a calcium/calmodulin-dependent protein phosphatase, is involved in movement, fertility, egg laying, and growth in *Caenorhabditis elegans*. *Mol. Biol. Cell* 13, 3281-3293

47. Campbell, B. E., Rabelo, E. M., Hofmann, A., Hu, M., and Gasser, R. B. (2010) Characterization of a *Caenorhabditis elegans glc s*even-like *p*hosphatase (*gsp*) orthologue from *Haemonchus contortus* (Nematoda). *Mol. Cell Probes* 24, 178-189

48. Ma, X., Zhu, Y., Li, C., Shang, Y., Meng, F., Chen, S., and Miao, L. (2011) Comparative transcriptome sequencing of germline and somatic tissues of the *Ascaris suum* gonad. *BMC Genomics* 12, 481

49. Hu, M., Campbell, B. E., Pellegrino, M., Loukas, A., Beveridge, I., Ranganathan, S., and Gasser, R. B. (2007) Genomic characterization of *Tv-ant-1*, a *Caenorhabditis elegans tag-61* homologue from the parasitic nematode *Trichostrongylus vitrinus*. *Gene* 397, 12-25

50. Ma, G. X., Zhou, R. Q., Hu, S. J., Huang, H. C., Zhu, T., and Xia, Q. Y. (2014) Molecular characterization and functional analysis of serine/threonine protein phosphatase of *Toxocara canis*. *Exp. Parasitol.* 141, 55-61

51. Chu, D. S., Liu, H., Nix, P., Wu, T. F., Ralston, E. J., Yates, J. R., III, and Meyer, B. J. (2006) Sperm chromatin proteomics identifies evolutionarily conserved fertility factors. *Nature* 443, 101-105

52. Jiang, D., Malone, J., Townsend, R., Weil, G. J., and Li, B. (2012) Multiplex proteomics analysis of gender-associated proteins in *Brugia malayi*. *Int. J. Parasitol.* 42, 841-850

53. Walter, R. D., and Albiez, E. J. (1981) Inhibition of NADP-linked malic enzyme from *Onchocerca volvulus* and *Dirofilaria immitis* by suramin. *Mol. Biochem. Parasitol.* 4, 53-60

54. Dunn, T. S., Raines, P. S., Barrett, J., and Butterworth, P. E. (1988) Carbohydrate metabolism in *Onchocerca gutturosa* and *Onchocerca lienalis* (Nematoda: Filarioidea). *Int. J. Parasitol.* 18, 21-26

55. Liang, R., Yu, W. D., Du, J. B., Yang, L. J., Yang, J. J., Xu, J., Shang, M., and Guo, J. Z. (2007) Cystathionine beta synthase participates in murine oocyte maturation mediated by homocysteine. *Reprod. Toxicol.* 24, 89-96

56. Kaur, J., and Kaul, G. (2008) Protein translocation pathways across the inner and outer mitochondrial membranes. *Indian Journal of Biochemistry & Biophysics* 45, 149-156

57. Billing, O., Kao, G., and Naredi, P. (2011) Mitochondrial function is required for secretion of DAF-28/Insulin in *C. elegans*. *PLoS One* 6, e14507

58. Choy, R. K., Kemner, J. M., and Thomas, J. H. (2006) Fluoxetine-resistance genes in *Caenorhabditis elegans* function in the intestine and may act in drug transport. *Genetics* 172, 885-892

59. Cinkornpumin, J. K., Wisidagama, D. R., Rapoport, V., Go, J. L., Dieterich, C., Wang, X., Sommer, R. J., and Hong, R. L. (2014) A host beetle pheromone regulates development and behavior in the nematode *Pristionchus pacificus*. *Elife.* 3, e03229

60. Thomas, J. H., Stern, M. J., and Horvitz, H. R. (1990) Cell interactions coordinate the development of the *C. elegans* egg-laying system. *Cell* 62, 1041-1052

61. Axang, C., Rauthan, M., Hall, D. H., and Pilon, M. (2007) The twisted pharynx phenotype in *C. elegans*. *BMC. Dev. Biol.* 7, 61

62. Burket, C. T., Higgins, C. E., Hull, L. C., Berninsone, P. M., and Ryder, E. F. (2006) The *C. elegans* gene *dig-1* encodes a giant member of the immunoglobulin superfamily that promotes fasciculation of neuronal processes. *Dev. Biol.* 299, 193-205

63. Benard, C. Y., Boyanov, A., Hall, D. H., and Hobert, O. (2006) DIG-1, a novel giant protein, non-autonomously mediates maintenance of nervous system architecture. *Development* 133, 3329-3340

64. Hewitson, J. P., Harcus, Y. M., Curwen, R. S., Dowle, A. A., Atmadja, A. K., Ashton, P. D., Wilson, A., and Maizels, R. M. (2008) The secretome of the filarial parasite, *Brugia malayi*: proteomic profile of adult excretory-secretory products. *Mol. Biochem. Parasitol.* 160, 8-21

65. Moreno, Y., and Geary, T. G. (2008) Stage- and gender-specific proteomic analysis of *Brugia malayi* excretory-secretory products. *PLoS Negl. Trop. Dis.* 2, e326

66. Bennuru, S., Semnani, R., Meng, Z., Ribeiro, J. M., Veenstra, T. D., and Nutman, T. B. (2009) *Brugia malayi* excreted/secreted proteins at the host/parasite interface: stage- and gender-specific proteomic profiling. *PLoS Negl. Trop. Dis.* 3, e410

67. Geary, J., Satti, M., Moreno, Y., Madrill, N., Whitten, D., Headley, S. A., Agnew, D., Geary, T., and Mackenzie, C. (2012) First analysis of the secretome of the canine heartworm, *Dirofilaria immitis*. *Parasit. Vectors* 5, 140
